# The Axial Organ and the Pharynx Are Sites of Hematopoiesis in the Sea Urchin

**DOI:** 10.3389/fimmu.2019.00870

**Published:** 2019-04-25

**Authors:** Preethi Golconda, Katherine M. Buckley, Caroline R. Reynolds, Jennifer P. Romanello, L. Courtney Smith

**Affiliations:** Department of Biological Sciences, George Washington University, Washington, DC, United States

**Keywords:** echinoderm, EdU, hematopoietic tissue, proliferation, transcription factors, gene regulatory network

## Abstract

**Background:** The location of coelomocyte proliferation in adult sea urchins is unknown and speculations since the early 1800s have been based on microanatomy and tracer uptake studies. In adult sea urchins (*Strongylocentrotus purpuratus*) with down-regulated immune systems, coelomocyte numbers increase in response to immune challenge, and whether some or all of these cells are newly proliferated is not known. The gene regulatory network that encodes transcription factors that control hematopoiesis in embryonic and larval sea urchins has not been investigated in adults. Hence, to identify the hematopoietic tissue in adult sea urchins, cell proliferation, expression of phagocyte specific genes, and expression of genes encoding transcription factors that function in the conserved regulatory network that controls hematopoiesis in embryonic and larval sea urchins were investigated for several tissues.

**Results:** Cell proliferation was induced in adult sea urchins either by immune challenge through injection of heat-killed *Vibrio diazotrophicus* or by cell depletion through aspiration of coelomic fluid. In response to either of these stimuli, newly proliferated coelomocytes constitute only about 10% of the cells in the coelomic fluid. In tissues, newly proliferated cells and cells that express SpTransformer proteins (formerly Sp185/333) that are markers for phagocytes are present in the axial organ, gonad, pharynx, esophagus, and gut with no differences among tissues. The expression level of genes encoding transcription factors that regulate hematopoiesis show that both the axial organ and the pharynx have elevated expression compared to coelomocytes, esophagus, gut, and gonad. Similarly, an RNAseq dataset shows similar results for the axial organ and pharynx, but also suggests that the axial organ may be a site for removal and recycling of cells in the coelomic cavity.

**Conclusions:** Results presented here are consistent with previous speculations that the axial organ may be a site of coelomocyte proliferation and that it may also be a center for cellular removal and recycling. A second site, the pharynx, may also have hematopoietic activity, a tissue that has been assumed to function only as part of the intestinal tract.

## Background

### Hematopoiesis

Hematopoiesis is the process in which a multipotent hematopoietic stem cell differentiates into one of potentially many terminally differentiated blood cell types (1). This process is tightly regulated by micro-environmental cues in hematopoietic tissues that include secreted molecules and cell surface receptors. Cell fate decisions are controlled by a series of gene regulatory networks (GRNs) composed of sets of transcription factors that regulate changes in gene expression associated phenotypic differentiation toward one cell type or another. Hematopoiesis is generally conserved among vertebrates with mouse and, more recently, zebrafish serving as primary model systems for understanding this differentiation process (2). In adult vertebrates, the rates of hematopoiesis and the release of new cells from hematopoietic tissues vary based on the immune state of the organism and the turnover of specific categories of immune cells during pathogen interactions or injury repair.

### Cell Proliferation Is Used to Identify Hematopoietic Tissues

Proliferation of immune cells in response to pathogen challenge and subsequent changes in immune cell populations has been noted for many invertebrate species (3–5). Hemocyte proliferation has been used to identify sites of hematopoiesis in insects, gastropods, bivalves, cephalopods, and crustaceans (6–9). Proliferative responses are consistent with a turnover of cells that participate in phagocytosis to clear microbes. In adult sea urchins, phagocytosis is carried out by the phagocyte class of coelomocytes (10) that decrease in concentration during clearance of bacteria injected into the coelom, and subsequently return to pre-challenge levels (11, 12). Coelomocyte proliferation is evident but low in non-challenged sea urchins as measured by ^3^H-thymidine incorporation (13) or BrdU incorporation (14). These experimental approaches demonstrate that coelomocyte proliferation can be measured and that a similar strategy may be employed to identify the hematopoietic tissues in echinoderms.

### The Innate Immune System in Adult Sea Urchins

The cellular innate immune system of the purple sea urchin, *Strongylocentrotus purpuratus*, is mediated by four morphologically distinct types of coelomocytes that include phagocytes, colorless, and red spherule cells, and vibratile cells [(15), reviewed in (16, 17)]. Phagocytes respond to immune challenges through phagocytosis (10), encapsulation, syncytia formation (18), and expression of complement components (19) and the *SpTransformer* (*SpTrf*, formerly *Sp185/333*) gene family (20–22), which encodes a large array of secreted, anti-pathogen SpTrf proteins (10, 23–27). In immune challenged larvae, *SpTrf* gene expression is restricted exclusively to filopodial blastocoelar cells (28) that are likely homologous to adult phagocytes. Thus, *SpTrf* gene expression and protein production are used here as a marker for phagocytes in the coelomic fluid (CF) and embedded in adult tissues (29).

Sea urchins down-regulate their immune response when they are maintained long term in artificial sea water in recirculating aquaria. This immunoquiescent (IQ) state includes decreased expression of at least some of their immune response genes (21, 22, 30) and reduced concentrations of coelomocytes in the CF (23, 31). Intracoelomic injection lipopolysaccharide (LPS) reverses the IQ state within 24 h resulting in a 7-fold increase in the number of coelomocytes in the CF, including a 10-fold increase in SpTrf^+^ phagocytes (23). Consequently, IQ sea urchins responding to challenge are optimal for tracking coelomocyte proliferation. In tissues from sea urchins responding to immune challenge, the axial organ shows notable increases in *SpTrf* expression, numbers of SpTrf^+^ cells, and levels of SpTrf proteins relative to other adult tissues (29). The axial organ is a small, bean shaped organ that is located along the central vertical axis of the oblate spheroid shaped adult echinoid and is associated with the stone canal, which is part of the water vascular system (32, 33). Since the early 1800s, speculations regarding its function have included the origin of coelomocytes, removal and degradation of coelomocytes and foreign cells, renal-like filtering and excretion, and cardiac-like activity that distributes fluid through the haemal system (13, 29, 33–45). Many of these hypotheses are based on histology and/or up-take of tracers and injected cells that have perpetuated the confusion about the functions of this organ.

### Identification of Hematopoietic Tissues Based on Expression of Genes Encoding Conserved Transcription Factors

The arms race between the host immune system and pathogens drives immune gene diversification and subsequent selection based on improved immune responses to pathogens [reviewed in (46)]. This process leads to rapid evolutionary changes in immune genes that encode pathogen recognition receptors (PRRs) or effector proteins, and this diversity makes it challenging to identify markers of shared and evolutionarily conserved aspects of immune responses among groups of animals. An example of gene diversification in regular echinoids is the *SpTrf* gene family, which is composed of duplicated and clustered genes that encode a wide range of similar but slightly different anti-pathogen proteins (26, 47). On the other hand, genes encoding proteins involved in signaling pathways that are likely induced by PRRs and associated regulatory transcription factors including those that function in GRNs tend to be more conserved over long periods of evolutionary time (48). In tetrapods, hematopoiesis occurs primarily in the thymus and bone marrow, although this process also occurs in unique hematopoietic organs in fish and birds; the head kidney and bursa of Fabricious, respectively. Despite these anatomical differences among vertebrates, these tissues express similar suites of homologous regulatory systems to control both hematopoietic tissue development and immune cell differentiation. Thus, comparative investigations of immune system development and cell differentiation have been used to understand fundamental aspects of hematopoiesis.

The use of conserved genes that function in the hematopoietic regulatory circuitry has been extended in comparative studies of invertebrate phyla to identify similarities in hematopoietic processes, and much is shared between vertebrates and non-vertebrates [reviewed in (49) and (50) and see references therein]. For example, in arthropods, the embryonic development of the hematopoietic tissue, the lymph gland (51–54), and the production of larval hemocytes employ transcription factors that are homologous to those in mammals [(55–57), reviewed in (6, 7)]. Adult *Drosophila* produce hemocytes from sessile hemocyte patches or hubs that are associated with the dorsal heart, and are analogous to peripheral hematopoiesis in mammals (54, 58). The homologous core regulatory system in both mammals and insects activate gene batteries that drive the differentiation and maturation of the immune components for both groups.

Because echinoderms are invertebrate deuterostomes and a sister phylum to the chordates, they are an important phylogenetic group in which to identify aspects of hematopoiesis that are evolutionarily conserved. Although this process is not well understood in adult sea urchins, immune cell development in the embryonic and larval stages relies on a GRN that shares commonalities with vertebrate hematopoiesis [(28, 59–61); reviewed in (16)]. These homologous transcription factors and transcriptional regulators including SpGatac (homologous to vertebrate Gata1/2/3; GATA core binding sequence), SpGcm (glial cells missing), SpScl (Scl/Tal1; T-cell acute lymphocytic leukemia protein 1), SpId (Id-1/2/3/4; inhibitor of DNA-binding/differentiation proteins), SpTCF (TCF/LEF; transcription factor/lymphoid enhancer-binding factor), Sp-E-protein (E2A/HEB/E2-2; transcription factor E2-Alpha), and SpLmo2 (Lmo2; LIM domain only 2). The larval immune response is mediated by two cell lineages that arise from a ring of non-skeletogenic mesenchyme (NSM) that is specified during mesenchyme blastula stage; pigment cells and a heterogeneous suite of blastocoelar cells (28, 59, 60). Pigment cells develop from cells located within the aboral NSM, whereas the oral NSM cells develop into blastocoelar cells. Pigment cell precursor specification begins with the expression of SpGcm which, along with other factors, activates a gene battery involved in immune response and pigment synthesis (62, 63). Terminally differentiated pigment cells express two enzymes that are involved in the synthesis of echinochrome A, an anti-bacterial naphthoquinone (64–66). Pigment cells also express *SpTep2* that encodes a complement-like thioester-containing protein and *SpSRCR142* that encodes a scavenger receptor (28). In contrast, during mesenchyme blastula stage, the oral NSM cells undergo a short period of pluripotency that coincides with expression of the transcription factor genes, *SpGatac* and *SpScl*. These cells differentiate later into four morphologically distinct types of blastocoelar cells identified as globular, filopodial, amoeboid, and ovoid cells (28). These subtypes express distinct immune effector genes in which *SpTrf* expression is restricted to filopodial cells and *SpMacpfA2* expression is restricted to globular cells. Although it is not known whether these transcription factors function in regulating hematopoiesis in adult sea urchins, a similar GRN may be deployed in the adult as in the larval stages to regulate coelomocyte proliferation and differentiation.

To characterize coelomocyte proliferation in adult sea urchins and to identify adult hematopoietic tissues, IQ animals were either challenged with the marine bacterial pathogen, *Vibrio diazotrophicus*, or coelomocyte concentration was reduced by partial CF depletion. Removing CF was used to simulate the effects of bacterial clearance by phagocytosis and/or encapsulation that correlates with transient decreases in coelomocyte concentration (11, 12). In agreement with previous reports (14, 67), newly proliferated coelomocytes are detected *in vivo* based on incorporation of ethynyl deoxyuridine (EdU) into DNA in response to both *V. diazotrophicus* and CF depletion. The appearance of newly proliferated cells corresponds with increased numbers of coelomocytes. The accumulation of newly proliferated coelomocytes is slow as is the turnover rate of at least some cells in the CF. Cell proliferation is also evident in the axial organ, gonad, gut, and esophagus, although the rate of cell proliferation is not affected by immune challenge vs. injury. However, both qPCR and RNAseq analysis of adult tissues indicate that the expression levels of genes encoding transcription factors that function in the larval hematopoiesis GRN are elevated in the axial organ and the pharynx compared to coelomocytes, gonads, esophagus, and gut. A broader analysis of transcript prevalence in adult tissues indicates that transcripts associated with apoptosis are enriched in the axial organ. This suggests that the axial organ may serve as a site of cell turnover in addition to hematopoiesis, which is in agreement with a previous report (44). The results presented here suggest that both the axial organ and the pharynx are echinoid hematopoietic tissues that function in the production and release of coelomocytes into the CF.

## Materials and Methods

### Animal Maintenance

Adult sea urchins, *Strongylocentrotus purpuratus*, were obtained from Marinus Scientific Inc. (Long Beach, CA) or the Southern California Sea Urchin Company (Corona del Mar, CA) and maintained in an aquarium facility at George Washington University in artificial sea water (Crystal Sea Marine Mix, Marine Enterprises). Aquarium water temperature was held at 14°C and 32–34‰ salinity with weekly water changes (5–10% volume). Sea urchins were housed in recirculating aquaria for at least 6 months prior to experimentation to ensure an IQ state (31). Animals were fed Welpac Dashi Kombu dried seaweed (FoodServiceDirect.com) that was rehydrated in sea water.

IQ sea urchins of similar sizes were assigned randomly to either the experimental or control groups. For each animal, the body volume (BV) in ml was estimated using the equation for an oblate spheroid

$$\begin{array}{l} BV=\left(\frac{4}{3}\pi \right)\cdot r^{2}\cdot \left(\frac{h}{2}\right) \end{array}$$

according to Elliot et al. (68) where *r* is the horizontal radius and *h* is the height (mm) of the test. For the animals used in this study, BV estimates ranged from 16 to 69 ml.

For some experiments that evaluated cell proliferation, sea urchins were collected from subtidal sites near Santa Barbara CA. These sea urchins were housed in the open sea water system at the University of California at Santa Barbara for 3 weeks prior to use and during the experimental time period, and were fed freshly collected kelp. They were assumed to be non-IQ.

### Immune Challenge and Coelomic Fluid Depletion

The marine bacterial species, *Vibrio diazotrophicus* [ATCC, item #33466; (69, 70)], was grown in Marine Broth (Difco) at room temperature according to Sherman et al. (24). Bacteria were heat-killed at 95°C for 30 min. To determine bacterial concentration, cells were pelleted, resuspended in standard phosphate buffered saline (PBS; 137 mM NaCl, 2.7 mM KCl, 4.3 mM Na_2_HPO_4_, 1.47 mM KH_2_PO_4_; pH 7.4), and stained with fluorescein isothiocyanate (FITC; 0.2 mg/ml; Fisher Scientific) for 30 min at 37°C. Cells were washed, resuspended in artificial coelomic fluid [aCF; 10 mM CaCl_2_, 14 mM KCl, 50 mM MgCl_2_, 398 mM NaCl, 1.7 mM Na_2_HCO_3_, 25 mM Na_2_SO_4_, pH 7.4 (22)] and counted using a hemocytometer on an Axioskop fluorescent microscope (Carl Zeiss Microscopy). Unstained bacteria were counted on an Accuri C6 flow cytometer (Benton Dickinson). Heat-killed bacteria were either stored at 4°C and used within a week or stored in 20% glycerol at −20°C in individual aliquots.

Coelomocyte proliferation was stimulated by removing 1.5% or 5% (once or twice) of the estimated BV from adult IQ animals by inserting a needle through the peristomial membrane and into the coelomic cavity and aspirating 0.24–3.45 ml of CF depending on animal size and percentage of the BV to deplete. CF (50 μl) was removed from control animals and diluted into an equal volume of an anticoagulant, calcium- and magnesium-free seawater with EDTA and HEPES [CMFSW-EH; 460 mM NaCl, 10.73 mM KCl, 7.06 mM Na_2_SO_4_, 2.38 mM NaHCO_3_, 70 mM EDTA, 20 mM HEPES; pH 7.4 (10, 18)] for the purposes of cell counts. Proliferation was evaluated based on the incorporation of EdU (25 mM in aCF; ThermoFisher), which was injected on day 0 into each animal through the peristomial membrane for final concentration of 0.18 mM based on BV estimates. IQ animals in the experimental group (*n* = 3) received 1 × 10^5^ *V. diazotrophicus*/ml BV 15 min after the EdU injection. Control animals (*n* = 3) received similarly estimated volumes of aCF. Injections of EdU and bacteria or aCF were repeated for a second experiment on days 1, 4, and 6. Cell counts were performed daily (days 0–15) after withdrawing 100 μl CF using a 26 gauge needle and a 1 ml syringe preloaded with 500 μl of ice cold CMFSW-EH. Samples were kept on ice and evaluated for nuclear incorporation of EdU or stored at −20°C and evaluated within a week. Total coelomocyte cell counts and differential phagocyte counts were performed using a hemocytometer or a TC20 automated cell counter (Bio-Rad).

Three groups of non-IQ sea urchins were injected with EdU as described above on days 0, 3, and 6. Group 1 (*n* = 2) received *V. diazotrophicus* on days 0, 3, 6, and 21, group 2 (*n* = 2) received *V. diazotrophicus* on days 0, 3 and 6 and aCF on day 21, and group 3 (*n* = 2) received aCF on days 0, 3, 6, and 21. On day 22, coelomocytes were collected by needle aspiration and diluted into CMFSW-EH as described above, incubated in prefix without Triton X-100 for 15 min at room temperature, and followed by fix without Triton-X100 for 15 min at room temperature according to Brockton et al. (23). Postfix in cold methanol was omitted. Fixed cells were washed several times with AC320 buffer [see (23)] and stored at 4°C. Sea urchins were sacrificed on day 22 and the axial organ and esophagus from each animal were collected, fixed using the same protocol as for coelomocytes followed by extensive washing in AC230 and storage at 4°C.

### Immunohistochemistry

Coelomocytes (10^5^ cells) from IQ sea urchins were centrifuged for 5 min at 1,000 × *g* at 4°C onto Superfrost Plus slides (Shandon; ThermoScientific) using three-slot chimneys and cytospin slide holders (Eppendorf) as described (10) and cells were fixed for immunofluorescence according to Brockton et al. (23). EdU was detected using an Alexa Fluor 555 Click-It EdU Imaging Kit (Invitrogen) according to the manufacturer's protocol. Cells on slides were incubated in freshly prepared blocking solution (3% BSA [Sigma-Aldrich], 3% normal goat serum [Fisher Scientific]) in standard PBS for 45 min at room temperature in a humid chamber. This was followed by incubation with monoclonal mouse anti-actin (MP Biomedicals, 1:500 dilution in blocking solution) and an equal mixture of rabbit anti-SpTrf-66, –68, –71 (1:4,000 dilution in blocking solution according to Brockton et al. (23) and Dheilly et al. (25) for 1 h at room temperature in a humid chamber. Slides were washed three times with PBS in a Coplin jar for 5 min each with intermittent agitation followed by incubation with a mixture of donkey anti-mouse IgG conjugated to Alexa Fluor 488 (Invitrogen, 1:200 dilution in blocking solution) and goat anti-rabbit IgG conjugated to Alexa Fluor 568 (Invitrogen, diluted 1:400 in blocking solution) for 45 min at room temperature in a humid chamber. Slides were washed as above and cells were mounted using Prolong Gold Anti-fade Reagent that included 4',6-diamidino-2-phenylindole dihydrochloride (DAPI; Invitrogen). Slides were stored at 4°C until evaluation and at −80°C for long term storage. Cells were observed in an Axioskop fluorescent microscope (Carl Zeiss Microscopy) and imaged with an FVII digital camera (Olympus) and Microsuite imaging software (Olympus) or with the LSM710 Upright confocal fluorescence microscope (Carl Zeiss Microscopy) and ZEN 2011 imaging software (Carl Zeiss Microscopy).

Fixed coelomocytes from non-IQ sea urchins were processed in solution with the Alexa Fluor 488 Click-It EdU Imaging Kit (Invitrogen) according to the manufacturer, counter stained with DAPI, and evaluated on an Accuri C6 Flow Cytometer (Benton Dickinson) to quantify EdU^+^ cells. Background levels were determined by omitting the Click-It kit reagents, which were subtracted from data after processing for EdU incorporation.

### Tissue Collection and Immunohistochemistry

IQ sea urchins injected with EdU and from which CF had been depleted were sacrificed on day 4 (1.5% CF depletion) or day 6 (5% CF depletion). Adult tissues were obtained after using scissors to bisect the test equatorially into oral and aboral hemispheres. The pharynx was dissected from within Aristotle's Lantern as described (29). Tissues were stored in RNA*later* (Ambion; ThermoScientific) at −80°C until used for gene expression analysis. For immunohistochemistry, tissues were immediately fixed and frozen according to Majeske et al. (18) followed by sectioning with a cryostat. Slides were processed immediately or stored at −20°C until needed. Slides were incubated in 100% methanol for 1 h at −20°C followed by rehydration in a Coplin jar for 20 min in PBS with 4% BSA with intermittent agitation. Immunohistochemistry and evaluation of EdU incorporation were carried out as described above for coelomocytes with minor modifications. Slides were incubated first in the Click-It Alexa Fluor Reaction Cocktail for 45 min followed by incubation in freshly prepared blocking solution (4% BSA and 4% normal goat serum in PBS) for 2 h at room temperature. The primary and secondary antibody incubations were extended to 80 min each.

The axial organ and esophagus from non-IQ sea urchins were collected and fixed on day 22. Tissues were sectioned in a cryostat as described above followed by drying on slides at 37°C overnight. Sections were incubated in cold methanol for 1 h at −20°C, dried, and stored at −20°C. Sections were pre-incubated in 5% Triton X-100 in PBS for 20 min, blocked in 2% BSA in PBS, processed for EdU incorporation with the Alexa Fluor 488 ClickIt kit for EdU (Invitrogen), counter stained with DAPI, and mounted in FlowFade AntiFade medium (Thermo Fisher Scientific). Sections were imaged with an Axioskop fluorescent microscope (Carl Zeiss Microscopy) equipped with an Infinity 3 color digital camera and the associated imaging suite (Lumenera). Images of tissues from all animals were evaluated for the percentage of EdU^+^ nuclei. Between 1,860 and 8,354 nuclei were counted for sections on each slide.

### RNA Isolation and Gene Expression Analysis

Tissues dissected from IQ sea urchins were either stored in RNA*later* or processed immediately. Tissues were disrupted and homogenized using sterile pestles and RNA was isolated with the PrepEase RNA Spin Kit (Affymetrix) according to the manufacturers' instruction and stored at −80°C. RNA quality and integrity were assessed using gel electrophoresis (1.0% agarose with standard buffers for DNA plus either 0.2 μg/ml ethidium bromide or 0.4X SYBR® Safe DNA gel stain; Life Technologies), and imaged on a ChemiDoc XRS+ with Image lab Software (Bio-Rad). cDNA was synthesized from total RNA (140–600 ng) using the SMARTScribe RT-PCR Kit (Clontech; Takara Bio) with random hexamers (Operon) according to the manufacturers' protocols. cDNA quality and genomic DNA contamination were assessed by the message vs. gene sizes of the *SpL8* amplicons [*SpL8* encodes a homolog of the human ribosomal protein L8; GenBank accession number R62029 (71) also called Sp-Rpl8 [gene SPU_010692] on www.echinobase.org]. PCR was performed using GoTaq Green Mastermix (Promega). Cycling conditions had an initial denaturation at 94°C for 2 min followed by 25 cycles of 94°C for 30 s, annealing at primer-dependent temperatures (Supplementary Table 1A) for 30 sec, 72°C for 1 min, a final extension of 72°C for 5 min, and a 4°C hold.

Quantitative RT-PCR (qPCR) was performed with the cDNA templates and primers for individual transcripts (Supplementary Table 1B) using Express SYBR GreenER™ qPCR Supermix with premixed ROX (ThermoScientific). Reactions were performed in duplicate on a real-time PCR thermocycler (Eppendorf) and analyzed using the Mastercycler Ep Realplex software (Eppendorf). Gene expression values were normalized to *SpL8* expression levels.

### Statistical Analyses

Unpaired *t*-tests were used to identify significant differences between days or animal groups for EdU^+^ nuclei and for SpTrf^+^ cells per DAPI-labeled nuclei per section between tissues. Both Quartile and τ tests were used to identify outlier gene expression data for qPCR results based on the internal standard, *SpL8*. Both tests showed that expression in one control for the axial organ was an outlier that was omitted from subsequent statistical analyses. qPCR results for expression of individual genes were compared within and among treatment groups, and among different tissues using unpaired *t*-tests. Subsequent analyses employed one-way ANOVA (Excel) and two-way non-parametric ANOVA (GraphPad Prism 5). Significance was set at *p* ≤ 0.05 for all tests, which, in some cases did not agree between *t*-tests and the more conservative ANOVA analyses.

### Bioinformatics and RNAseq Analysis

RNAseq reads from adult sea urchin tissues were obtained from GenBank [SRA Project PRJNA81157 (72)] and mapped against the *S. purpuratus* genome (v4.2; www.echinobase.org) using Bowtie 2, v.2.3.3 (73). Read counts per transcript were quantified using SeqMonk (www.bioinformatics.babraham.ac.uk/projects/seqmonk/) and converted to counts per million (CPM) using edgeR (74). Heatmaps were generated in R using the pheatmap package (75). Gene ontology enrichment analysis was performed using the PANTHER Classification System (76).

## Results

### Newly Proliferated Coelomocytes Appear in the CF Following Immune Challenge

**Figure 1:**
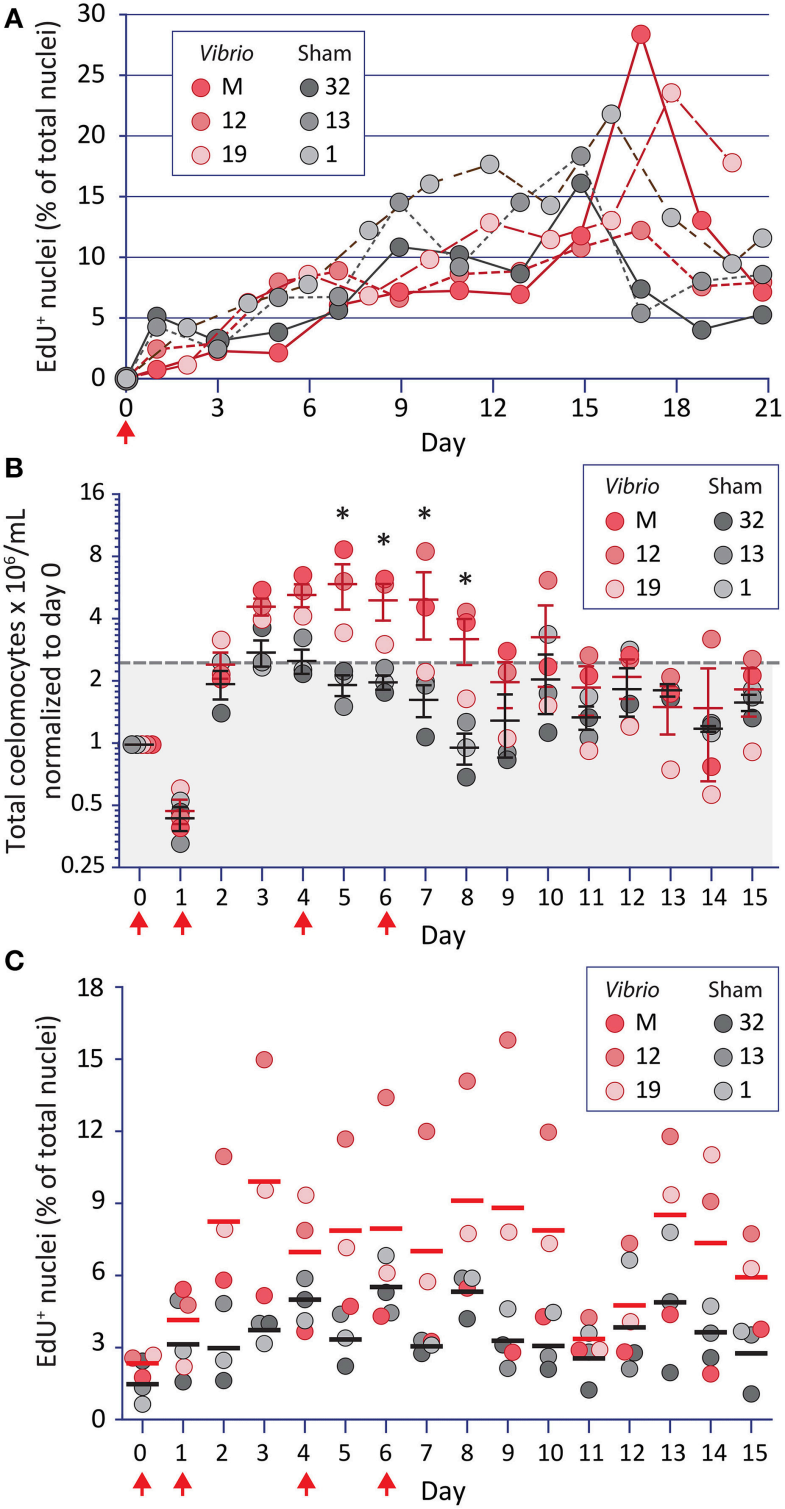
Increases in coelomocyte concentration and proliferation is induced by *Vibrio diazotrophicus* and tracked by EdU uptake. **(A)** EdU injected on day 0 (arrow) is incorporated into the DNA of phagocytes. Animals were also injected on day 0 with either heat-killed *Vibrio diazotrophicus* or aCF (sham injection). To reduce injury stress, CF was collected on alternating days. EdU incorporation is noted for phagocytes from both groups of sea urchins. **(B)** The same animals were challenged 54 days after the termination of the experiment shown in **(A)**. They received four injections (arrows) of EdU plus either heat-killed *V. diazotrophicus* or aCF (sham) and were evaluated for coelomocyte concentration. Animals that received *V. diazotrophicus* show up to a four-fold increase in coelomocyte concentration compared to the sham injected controls. Differences in cells per ml between groups are significant (^*^, *p* < 0.05) on days 5-8. The dashed gray line and gray shading indicate 2.2-fold higher cell concentration relative to day 0. **(C)** The cells from animals challenged with four injections of either *V. diazotrophicus* or aCF were evaluated for EdU incorporation. EdU^+^ cells are present on day 0, which was 75 days after the first injection of EdU given in the experiment shown in **(A)**. Challenge with *V. diazotrophicus* resulted in coelomocytes with a higher range of EdU^+^ nuclei than the sham injected controls.

Immunoquiescent (IQ) sea urchins decrease the concentration of coelomocytes in the CF (31). This state of IQ is reversed quickly in response to immune challenge with increases in coelomocyte concentration with a subsequent shift in the relative proportions of the phagocyte subtypes (23, 29). Hence, IQ sea urchins were used to determine whether increases in coelomocyte concentration involved cell proliferation or migration of tissue-resident coelomocytes into the CF. Six animals were injected once with EdU (67) plus either heat-killed *V. diazotrophicus* or an equal volume of aCF as the sham control and coelomocytes were collected and enumerated over 21 days. Results showed that although the coelomocyte concentration was variable (variation of 1.63-fold among samples), both groups exhibited a moderate increase of coelomocytes by day 3 post injection (Supplementary Figure 1A). Furthermore, some of this increase was due to cell proliferation (12% to 28% of glass-adherent phagocytes were EdU^+^; Figure 1A; Supplementary Figure 1B). Non-adherent coelomocytes were not evaluated with this approach [for a review of echinoderm coelomocytes, see (16)]. No differences were observed in cell concentration or proliferation between the immune-challenged vs. injury control groups, suggesting that a single injection of heat-killed *V. diazotrophicus* was insufficient to stimulate an immune response in IQ animals. However, this experiment demonstrated that EdU uptake and incorporation could identify newly proliferated coelomocytes and that these cells accumulated slowly in the CF.

To avoid the confounding effects of the significant genetic diversity among *S. purpuratus* individuals that are collected from wild, outbred populations (77, 78), the same sea urchins that received a single injection of EdU and a single challenge (Figure 1A, Supplementary Figure 1A) were used for a second analysis 54 days after the termination of the first experiment. To amplify the immune responses, sea urchins received four intracoelomic co-injections of EdU and either heat-killed *V. diazotrophicus* or aCF (sham control). Coelomocyte concentration was analyzed daily by monitoring small volumes of CF withdrawn over 15 days (Figure 1B). Animals challenged with *V. diazotrophicus* maintained significantly higher (*p* < 0.05) coelomocyte concentrations in the CF on days 5 through 8 compared to the sham injected counterparts. Coelomocyte concentrations returned to pre-injury levels by day 9 for immune challenged sea urchins, and by day 5 for the sham controls. These results agreed with previous reports (23, 29) and indicated that, in response to sufficient bacterial challenge, adult sea urchins increased the coelomocyte concentration in the CF earlier, longer, and to a greater extent than to injury responses alone.

Phagocyte proliferation was also assayed in this experiment (Figure 1C). At the beginning of the experiment, and prior to receiving four injections of EdU, animals showed low levels of EdU^+^ cells (0.6–2.7% on day 0) (Figure 1C). Given that 75 days had elapsed since the initial EdU injection, this indicated that at least some adult phagocytes were very long-lived. Within two days following the first injection of *V. diazotrophicus*, sea urchins exhibited higher proportions of EdU^+^ coelomocytes compared to sham injected controls. There was an average of 7.4% EdU^+^ phagocytes in the immune challenged animals on days 2 through 15 vs. 3.8% in controls. Although few of the time points reached statistical significance between the groups, the general trend indicated that immune challenged animals had higher proliferation of phagocytes than the controls. However, the maximum numbers of EdU^+^ phagocytes were lower than those observed when animals received a single injection (28% vs. 15.8%; compare Figures 1A,C). This may reflect a more rapid rate of phagocyte turnover perhaps as a consequence of the immune challenge driving increased clearance of the heat-killed *V. diazotrophicus*. When phagocyte proliferation was taken into account relative to increases in coelomocyte concentration in animals responding to immune challenge or injury, only about 10% of this change was likely due to proliferation. Thus, even though the phagocyte population is about 70% of the total coelomocytes [(15), reviewed in (16, 17)], results suggested that the expanded coelomocyte population size was mostly a consequence of the migration of tissue-resident coelomocytes into the CF.

### CF Depletion Induces Proliferation of Several Types of Coelomocytes

**Figure 2:**
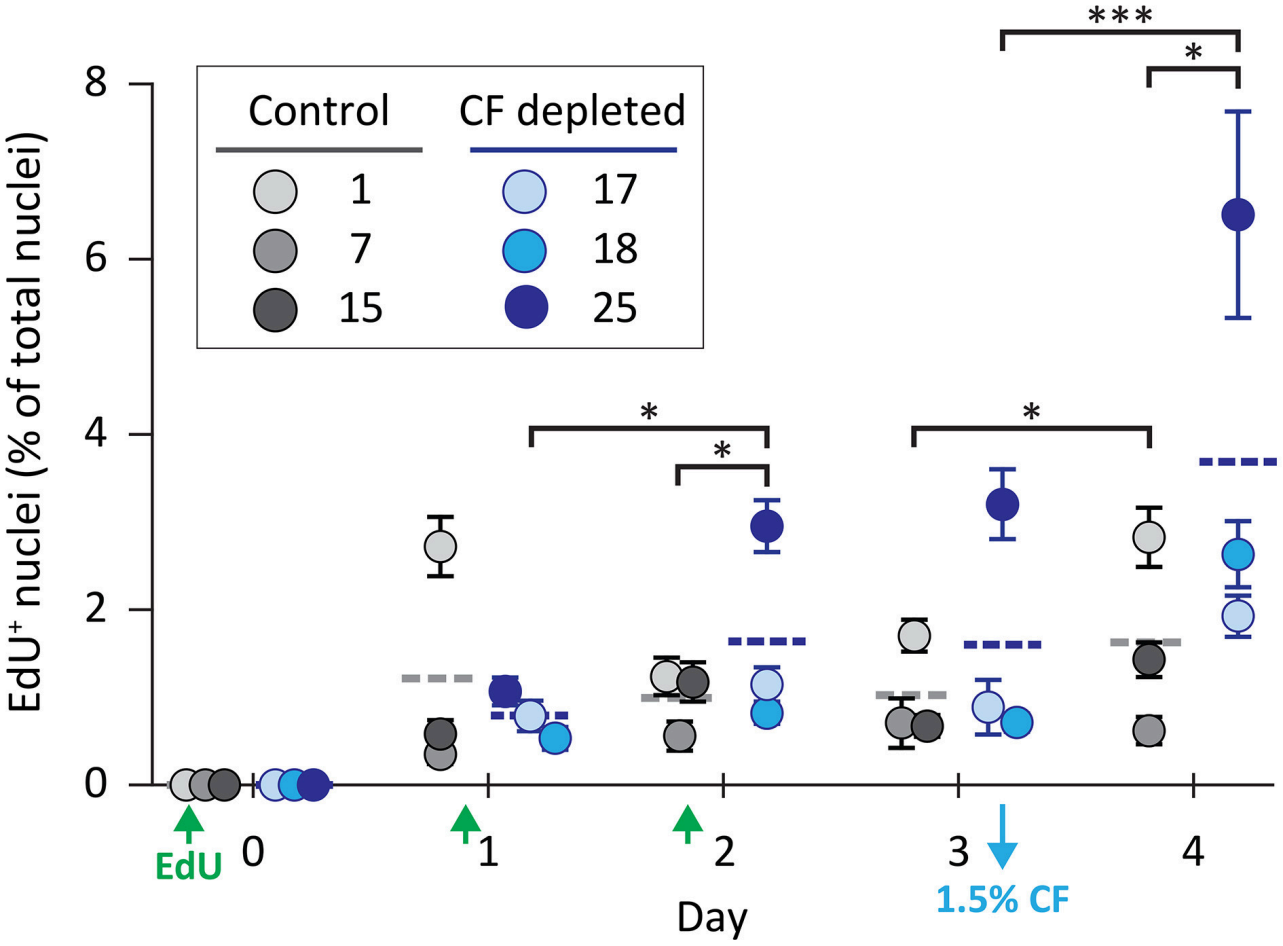
Coelomocyte proliferation is induced by CF depletion. Sea urchins were injected three times with EdU (green arrows) prior to the depletion of 1.5% of the CF (based on estimates of BV) on day 3 (blue arrow). CF was not depleted from the control animals. CF depletion resulted in a significant increase in the percentage of EdU^+^ coelomocytes on day 4 compared to the controls. Horizontal dashed lines indicate the means for each group. Brackets indicate paired groups with significant differences; ^*^, *p* < 0.01; ^***^, *p* < 0.0001.

As an alternative strategy to induce coelomocyte proliferation, CF was depleted from adult animals by aspiration (either 1.5%, 5% once, or 5% twice based on the estimated BV) to induce cell replacement by proliferation. Prior to CF depletion, animals were injected with EdU daily for 3 days to label new coelomocytes. During this time, there were few differences in EdU incorporation observed between the experimental and control groups on days 1, 2 (Figure 2). However, sea urchins that had 1.5% of their CF depleted on day 3 showed a significant (*p* < 0.0001) increase in EdU^+^ phagocytes on day 4 compared to non-depleted controls. When the experiment was repeated over 6 days after 5% CF depletion on day 0, the percentage of EdU^+^ phagocytes increased to an average of 11% by day 6 (results not shown). These results indicated that CF depletion could stimulate coelomocyte proliferation.

**Figure 3:**
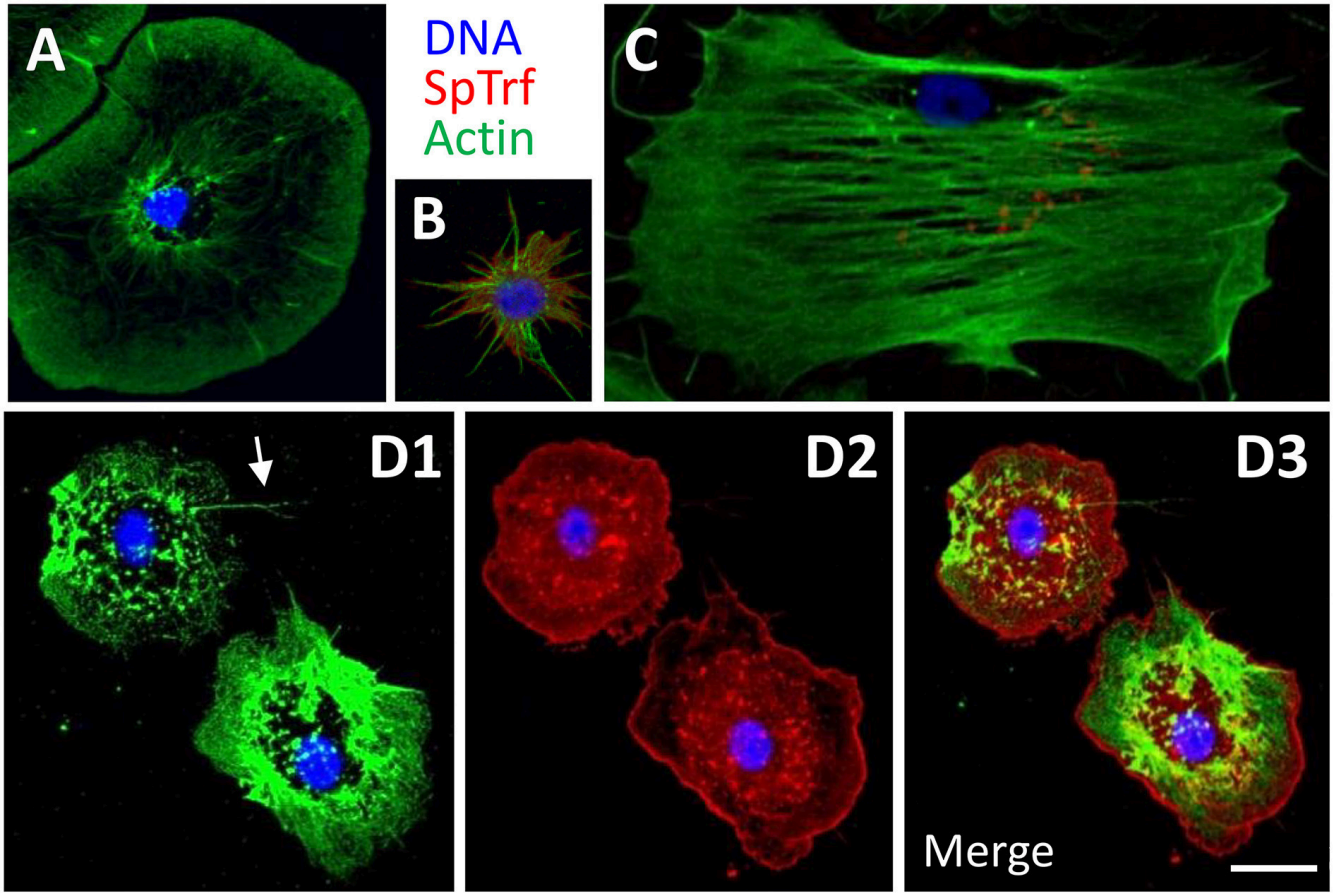
The phagocyte class of coelomocytes expresses SpTrf proteins. **(A)** Discoidal phagocytes have radially arranged actin cables and disc shaped cytoskeletal morphology. They show SpTrf expression rarely (83). **(B)** Small phagocytes are smaller than the large discoidal and polygonal phagocytes and are typically observed in filopodial morphology. A subset of small phagocytes shows high levels of SpTrf proteins [see also (23)]. **(C)** Polygonal phagocytes have actin cables that transverse the cell and define the polygonal shape of these cells. Subsets of polygonal phagocytes have SpTrf proteins localized to vesicles that are often positioned in a perinuclear location. **(D)** Medium phagocytes are defined by their intermediate size, between that of large and small phagocytes, and distinct cytoskeletal morphology of somewhat pentagonal shape with a few thin filopodia (arrow in D1). These cells show high levels of SpTrf proteins throughout the cell (D2, see also Supplementary Figure 2). Scale bar in D3 indicates 10 μm and applies to all figures.

Adult sea urchins have three morphologically distinct types of phagocytes with distinct sizes and cytoskeletal morphologies; discoidal, small, and polygonal (Figures 3A–C) (23, 79–82). Cytoskeletal actin cables transverse polygonal phagocytes, are arranged radially in discoidal phagocytes, and maintain a filopodial morphology in small phagocytes. To characterize the cell type repertoires in CF depleted animals, immunohistochemistry was used to differentiate among the actin-based cytoskeletal morphologies and to localize SpTrf proteins for the three phagocyte subtypes (Figures 3A–C) (23, 80, 81, 83). In the course of this work, an additional phagocyte subtype was identified (Figure 3D). These “medium” phagocytes were named was based on their cell size (20–30 μm in diameter), which was smaller than the polygonal and discoidal cells (30–50 μm), but larger than the small phagocytes (3–10 μm cell body excluding filopodia). Medium phagocytes tended to be hexagonal in shape when spread on glass and many exhibited fine filopodia (Figure 3D1, arrow; Supplementary Figure 2). These cells had an actin filament mesh condensed toward the cell periphery with little perinuclear actin organization. Notably, medium phagocytes expressed high levels of SpTrf proteins that were localized in a punctate pattern toward the center of the cells consistent with perinuclear vesicles, whereas at the cell periphery the SpTrf staining appeared evenly spread, perhaps in the cytoplasm (Figure 3D2). The peripheral localization was unlike other types of phagocytes in which SpTrf proteins are localized in vesicles or are present on the cell surface (Figures 3A–C) (23, 84). Because medium phagocytes have not been reported previously, it is not known whether their appearance was directly correlated with or induced by CF depletion.

**Figure 4:**
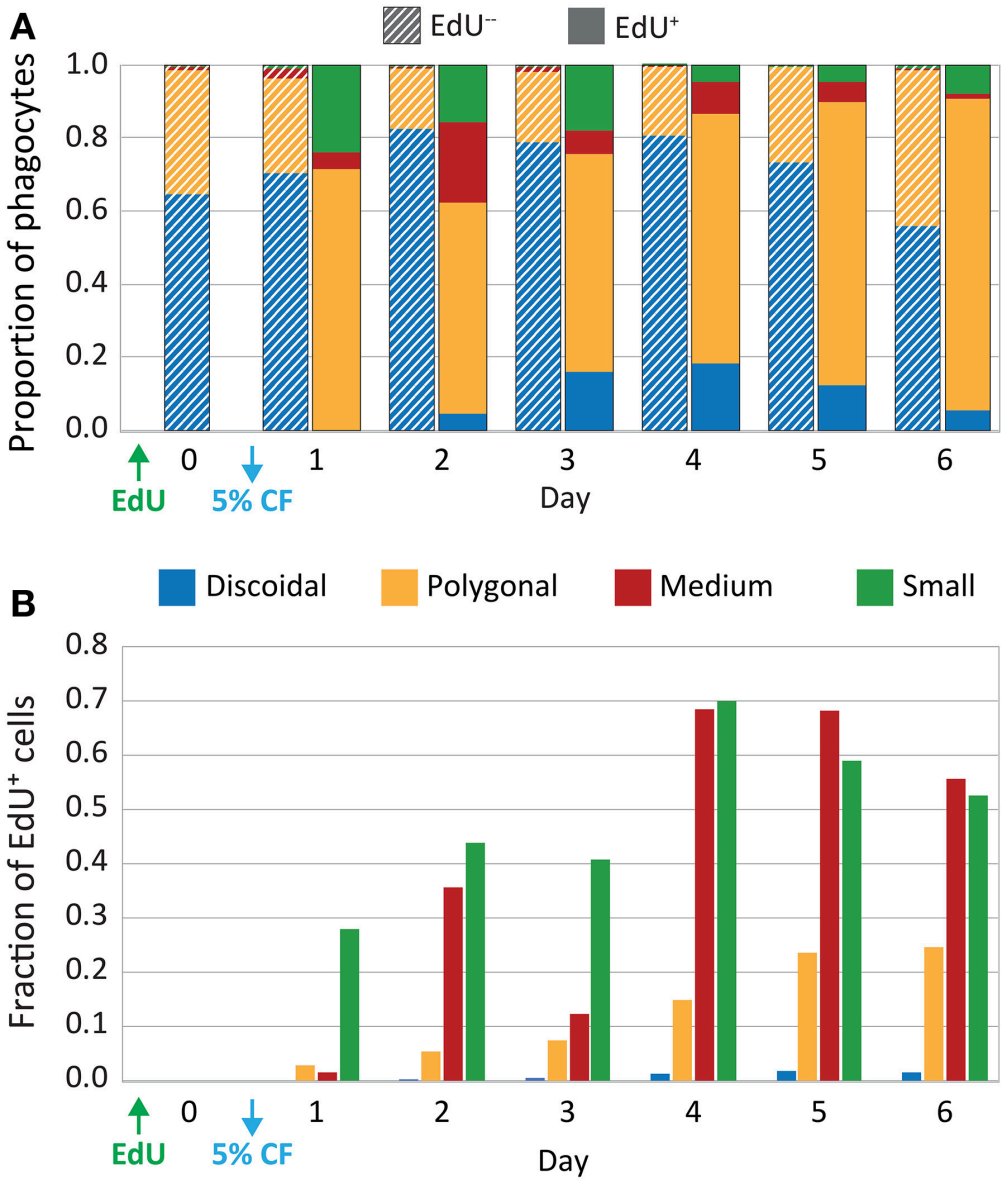
CF depletion induces proliferation of phagocytes. Sea urchins (*n* = 6) were injected with EdU and 5% of the estimated BV was depleted by CF aspiration. CF was collected from each animal on days 0–6, and phagocytes were processed for immunofluorescence to determine phagocyte type based on size, cytoskeletal morphology, SpTrf expression, and EdU incorporation. The resulting dataset of differential cell counts of 1,000 cells/sample/animal/day was used for the analyses that are displayed in both panels. **(A)** The EdU^−^ phagocyte types show minor variations in the proportions of each cell type over time. Discoidal cells are present at higher levels than polygonal cells and both are present at higher levels than the other phagocyte types. The EdU^+^ phagocytes show increases in all types of phagocytes, with the most notable increases in the polygonal, medium, and small phagocytes. **(B)** EdU incorporation for each phagocyte type is shown as the fraction of the total number of cells of each type. Both medium and small phagocyte populations show elevated EdU incorporation.

Cytoskeletal morphology was used in combination with EdU incorporation to quantify cell proliferation of different phagocyte subtypes in six sea urchins after a single 5% CF depletion. On day 0, prior to depletion, polygonal and discoidal phagocytes constituted the majority of the phagocytes (65 and 34%, respectively), whereas the small and medium phagocytes were rare (<2%, Figure 4A; however, see Supplementary Table 2), which was consistent with a previous report (23). Following CF depletion, the relative proportions of the phagocyte subtypes remained relatively consistent among the EdU^−^ populations over 6 days. However, among the EdU^+^ coelomocytes, the small and medium phagocytes made up a larger proportion of the newly proliferated cells. On day 2, the small and medium phagocytes constituted 38% of EdU^+^ cells vs. only 4% of the EdU^−^ population (Figures 4A,B; Supplementary Table 3). From day 2 to 6, significant fractions of small and medium phagocytes were EdU^+^ after CF depletion (Figure 4B; Supplementary Table 3). Thus, although all phagocyte types proliferated in response to CF depletion, the newly proliferated populations tended to be enriched for small and medium phagocytes.

### Cell Proliferation in Tissues to Identify Sites of Hematopoiesis

**Figure 5:**
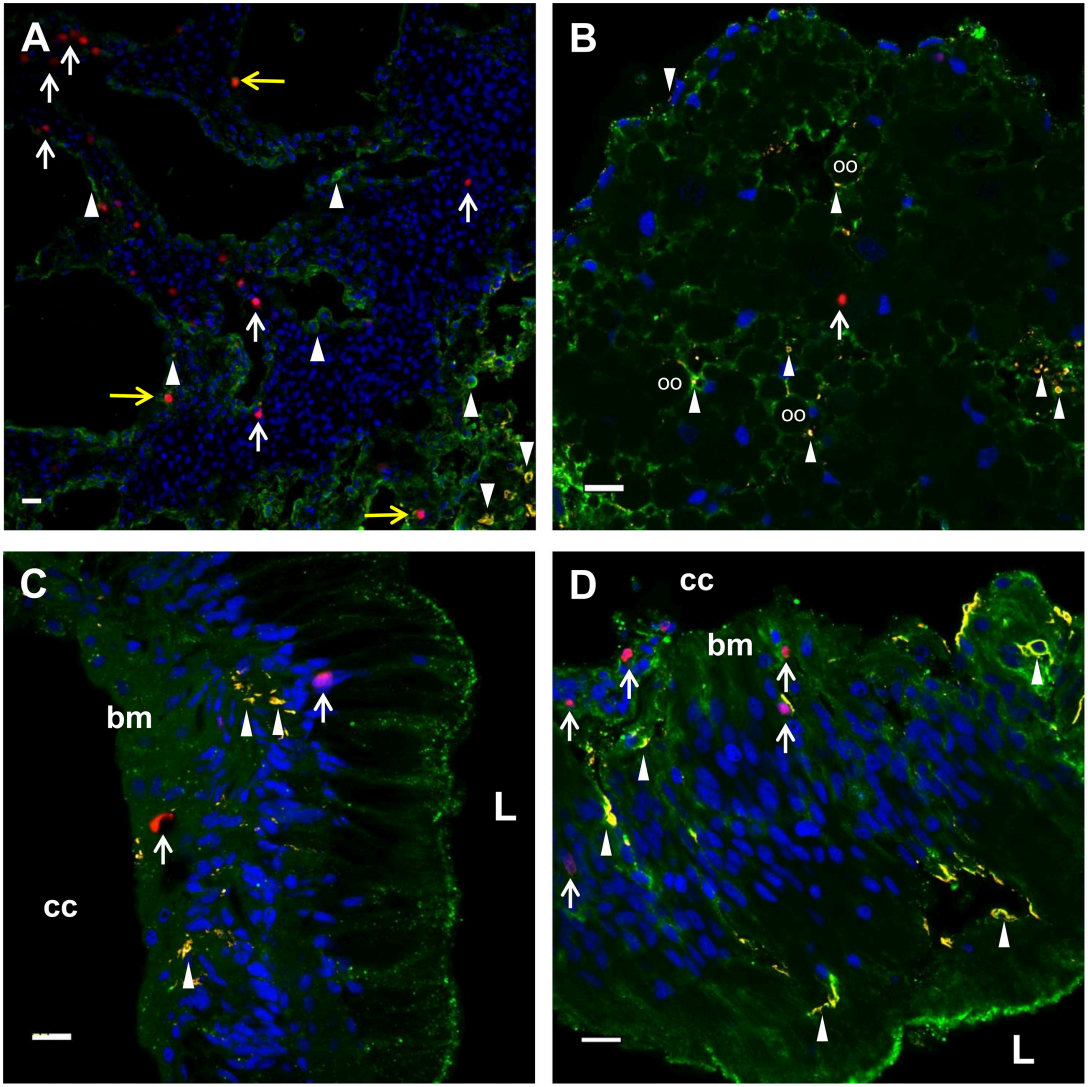
EdU^+^ and SpTrf^+^ cells are present in the axial organ, ovary, esophagus, and gut. **(A)** A transverse section of the axial organ shows EdU^+^ cells (white arrows) throughout the tissue. SpTrf^+^ cells (arrowheads) and EdU^+^SpTrf^+^ cells (yellow arrows) are also present. **(B)** A transverse section of the ovary shows EdU^+^ cells (arrows) and SpTrf^+^ cells (arrowheads) dispersed throughout the ovary. SpTrf proteins are also localized along the periphery of the oocytes (oo) as reported previously (29). **(C)** A longitudinal section of the esophagus shows EdU^+^ cells (arrows) among the columnar epithelial cells that line the lumen (L) and near the basement membrane (bm) that borders the coelomic cavity (cc). **(D)** A longitudinal section of the gut shows EdU^+^ cells (arrows) within the columnar epithelia as well as near the basement membrane (bm) that that faces the coelomic cavity (cc). SpTrf^+^ cells (arrowheads) are dispersed throughout the columnar epithelium, as reported previously (29). Sections are stained for DNA (DAPI, blue), actin (green), EdU (red), and SpTrf (yellow). See Supplementary Figure 3 for images of these sections prior to merging. Scale bars indicate 10 μm.

Adult IQ sea urchins subjected to either immune challenge or CF depletion have newly proliferated coelomocytes in the CF (Figures 1–3, Supplementary Figure 1). However, coelomocytes collected from echinoderms have never been observed in the process of mitosis (13) and do not proliferate in primary cultures [(85, 86); LCS, personal observations] presumably because they are terminally differentiated cells. Hence, the hematopoietic stem cells and progenitor cells that proliferate and differentiate are likely resident in hematopoietic tissues. To identify sites of hematopoiesis in *S. purpuratus*, EdU incorporation was evaluated in tissues (axial organ, esophagus, gut, and gonad) collected from animals 4 days after 1.5% CF depletion or 6 days after 5% CF depletion. In these experiments, SpTrf expression served as a marker for differentiated phagocytes (29). EdU^+^ cells were dispersed throughout the axial organ in CF depleted sea urchins (Figure 5A, white arrows; Supplementary Figure 3). SpTrf^+^ cells were primarily located along the linings of this porous tissue (Figure 5A; arrowheads) in agreement with previous findings (29). EdU^+^SpTrf^+^ cells were dispersed throughout the axial organ (Figure 5A, yellow arrows). In the ovary, EdU^+^ cells were dispersed throughout the tissue (Figure 5B, arrows), and SpTrf^+^ cells tended to be located at the periphery of oocytes (Figure 5B, arrowheads), in agreement with Majeske et al. (29). Depending on the sperm maturity in the testes among animals, the majority of sperm were either EdU^+^ or none were labeled [not shown, but see (29)]. In the esophagus and gut, EdU^+^ cells were present near the basement membrane and along the luminal side of these intestinal tissues (Figures 5C,D). SpTrf^+^ cells were dispersed along the both the basement membrane and within the columnar epithelia. SpTrf proteins were either present throughout the cytoplasm of cells within the columnar epithelia or were unevenly distributed in patches (Figures 5C,D, arrowheads). There was no identifiable pattern for EdU^+^SpTrf^+^ cells in the esophagus and gut.

In addition to the locations of EdU^+^ and SpTrf^+^ cells, their percentages were evaluated for tissues. For the groups of sea urchins from which CF was depleted (0%, 1.5%, 5% once or twice), all tissues showed highly variable percentages of EdU^+^ cells (Supplementary Figure 4A). Similar results were obtained for SpTrf^+^ and EdU^+^SpTrf^+^ cells (Supplementary Figures 4B,C). Sea urchins from which 5% of the CF was depleted twice showed lower levels of EdU^+^SpTrf^+^ cells in all tissues, which may reflect a stress response from depleting too much CF. Although CF depletion was not expected to show significant changes in the numbers of EdU^+^ cells in gonad, esophagus, or gut because they were not predicted to be locations of hematopoiesis, the axial organ also did not show a significant increase in proliferation in response to CF depletion. The identification of proliferated cells expressing SpTrf proteins (Supplementary Figure 4C) in any tissue may also be due to the influx of coelomocytes that proliferated elsewhere. Furthermore, increased numbers of newly proliferated cells may not be observed in any of the tissues if a steady state of coelomocyte release into the CF replaces those that were removed experimentally. Hence, interpretable results from CF depletion to identify the site of cell proliferation in a tissue may be masked by coelomocyte movement among tissues via the CF.

In an additional approach to quantify cell proliferation in the axial organ compared to the esophagus, which was used as the control tissue, non-IQ sea urchins housed in an open sea water system were injected with EdU and heat-killed *V. diazotrophicus* on days 0, 3, and 6 to induce cell proliferation in response to bacterial clearance. On day 21, sea urchins received either heat-killed *V. diazotrophicus* or aCF, whereas control animals received EdU and aCF on days 0, 3, 6, and aCF on day 21. Newly proliferated cells were distributed throughout the axial organ including the stone canal (Supplementary Figures 5A,B), however, the numbers of EdU^+^ cells in axial organ sections from animals in the three groups were not significantly different (Supplementary Table 4). The EdU^+^ nuclei in the esophagus showed a similar percentage of newly proliferated cells with no differences among groups (Supplementary Table 4; Supplementary Figure 4C). Coelomocytes collected from all non-IQ sea urchins and analyzed by flow cytometry indicated that the numbers of EdU^+^ nuclei were not different among the three groups (Supplementary Table 4). These results demonstrated that non-IQ sea urchins recently collected from their natural marine habitat and housed in an open sea water system had higher levels of newly proliferated cells in the axial organ, esophagus, and coelomocytes compared to IQ animals under long term housing in closed aquaria. Furthermore, injections of heat-killed *V. diazotrophicus* into non-IQ animals did not alter the level of cell proliferation compared to the controls.

### Genes Encoding Hematopoietic Transcription Factors and SpTrf Show Elevated Expression in the Axial Organ and the Pharynx

**Figure 6:**
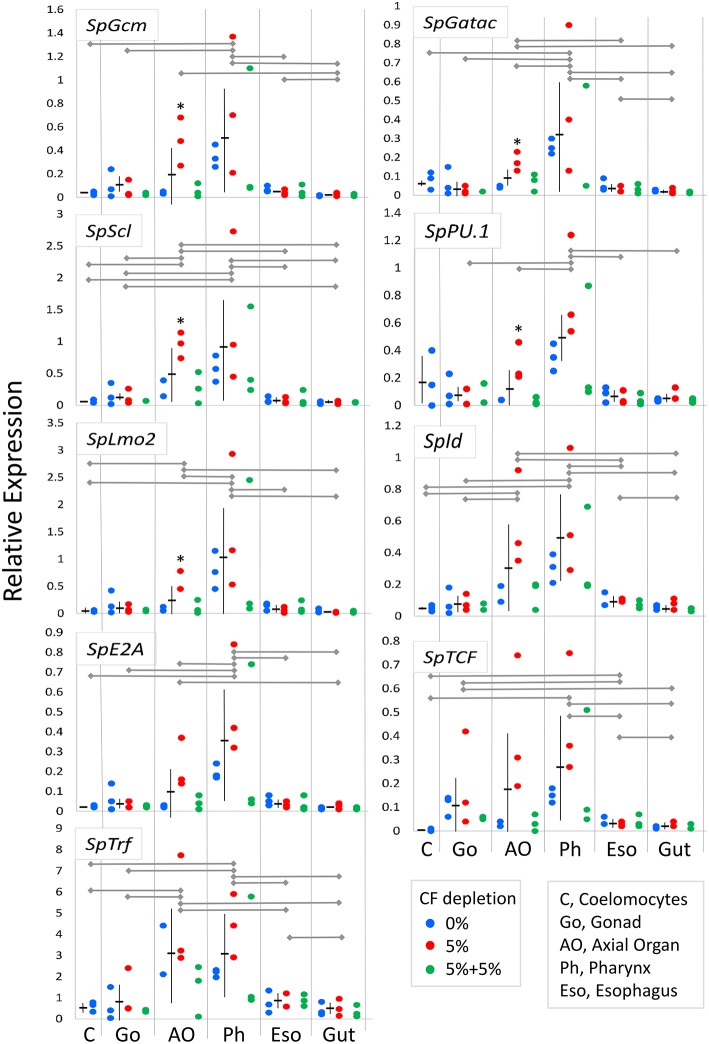
Expression of genes encoding transcription factors that regulate hematopoiesis are elevated in the axial organ and the pharynx. Expression of genes encoding hematopoiesis transcription factors in tissues and coelomocytes relative to the expression of *SpL8* that encodes a homolog of the large ribosomal protein L8 was based on qPCR results. Data from groups of animals from which CF was depleted (0%, 5% once or twice) were combined and compared between tissues by unpaired *t*-test and indicate that gene expression is highest in the pharynx with similar or somewhat lower expression in the axial organ. In some cases, gene expression in individual tissues shows significant increases in animals from which 5% CF was depleted (^*^) (see Supplementary Table 5). Expression of the *SpTrf* genes, which are markers for phagocytes, is highest in the axial organ and the pharynx. Horizontal gray lines for each gene indicate significant differences in gene expression between tissues (*p* < 0.05) based on unpaired *t*-tests (Supplementary Table 6) or a Bonferroni post-test after two-factor, non-parametric ANOVA (Supplementary Table 8). Mean expression ± standard deviation are shown in horizontal and vertical black lines for each tissue.

Coelomocytes are present throughout all adult tissues in *S. purpuratus* in addition to the CF and the fluid of the water vascular system (29, 36, 38) in which the red spherule cells can be observed as they circulate through the tube feet of *S. purpuratus* (LCS, personal observation). However, protein markers that identify all coelomocyte subsets are generally unknown and/or reagents are few (87). Consequently, it is difficult to demonstrate whether newly proliferated cells within tissues are coelomocytes, or, if they are coelomocytes, that they proliferated in the tissues in which they are observed. Thus, an alternative strategy to identify hematopoietic tissues in adult sea urchins was undertaken to quantify expression levels of genes that regulate immune cell differentiation and the development of the larval immune system (28, 59, 60). When expression was evaluated for transcription factor genes in tissues from within groups of animals from which CF was depleted (5% once or twice) or from the controls (0% depletion), few differences in gene expression were noted for most tissues (Supplementary Table 5). Exceptions were noted for five of nine genes in the axial organ for animals from which 5% of the CF was depleted once (Figure 6, asterisks). In general, these results suggested that the expression levels of genes in tissues were either not affected by coelomocyte replacement of depleted cells, or that the mRNA level could not be altered based on the balance of transcription vs. transcript recycling. When data for three CF depletion groups were combined, significant differences in gene expression were noted among tissues (Figure 6, Supplementary Table 6). More detailed analysis of gene expression among tissues indicated that 0% and 5% (once) CF depletion groups were the basis for these differences (Supplementary Table 7). Results showed that the axial organ and the pharynx had significantly higher expression levels for many of the genes encoding hematopoietic transcription factors compared to coelomocytes, gonad, esophagus, and gut (Figure 6; Supplementary Tables 6–8). *SpGcm* expression was significantly higher in the pharynx compared to all other tissues, whereas increased expression was only evident in the axial organ relative to gut (Figure 6; Supplementary Tables 6, 8). *SpGatac, SpScl*, and *SpLmo2* function together to regulate the differentiation of blastocoelar cells in late gastrulae, and all showed elevated expression in the pharynx and the axial organ relative to most other tissues. Significantly elevated expression of *SpId* was observed in both the pharynx and the axial organ relative to the other tissues. Elevated *SpTCF* expression was noted in the pharynx, however there were significant differences among many tissues. *SpE2A* expression was significantly elevated in the pharynx and the axial organ compared to most other tissues, whereas elevated expression of *SpPU.1* was observed only in the pharynx. Expression levels for all of the transcription factor genes were generally low in differentiated coelomocytes, esophagus, and gut. These results suggested that axial organ and particularly the pharynx may both function as sites of hematopoiesis in the adult sea urchin.

The expression of the *SpTrf* gene family was evaluated to determine whether the presence of phagocytes correlated with expression patterns of the transcription factors that control hematopoiesis. *SpTrf* gene expression was significantly higher in both the axial organ and the pharynx compared to all other tissues (Figure 6; Supplementary Tables 6, 8). Elevated *SpTrf* expression in the axial organ has been reported previously (29). This result indicated a correlation between tissue-resident phagocytes and elevated expression of hematopoietic transcription factors, which was consistent with the axial organ and the pharynx as possible sites of phagocyte differentiation and in which the phagocytes may tend to remain.

**Figure 7:**
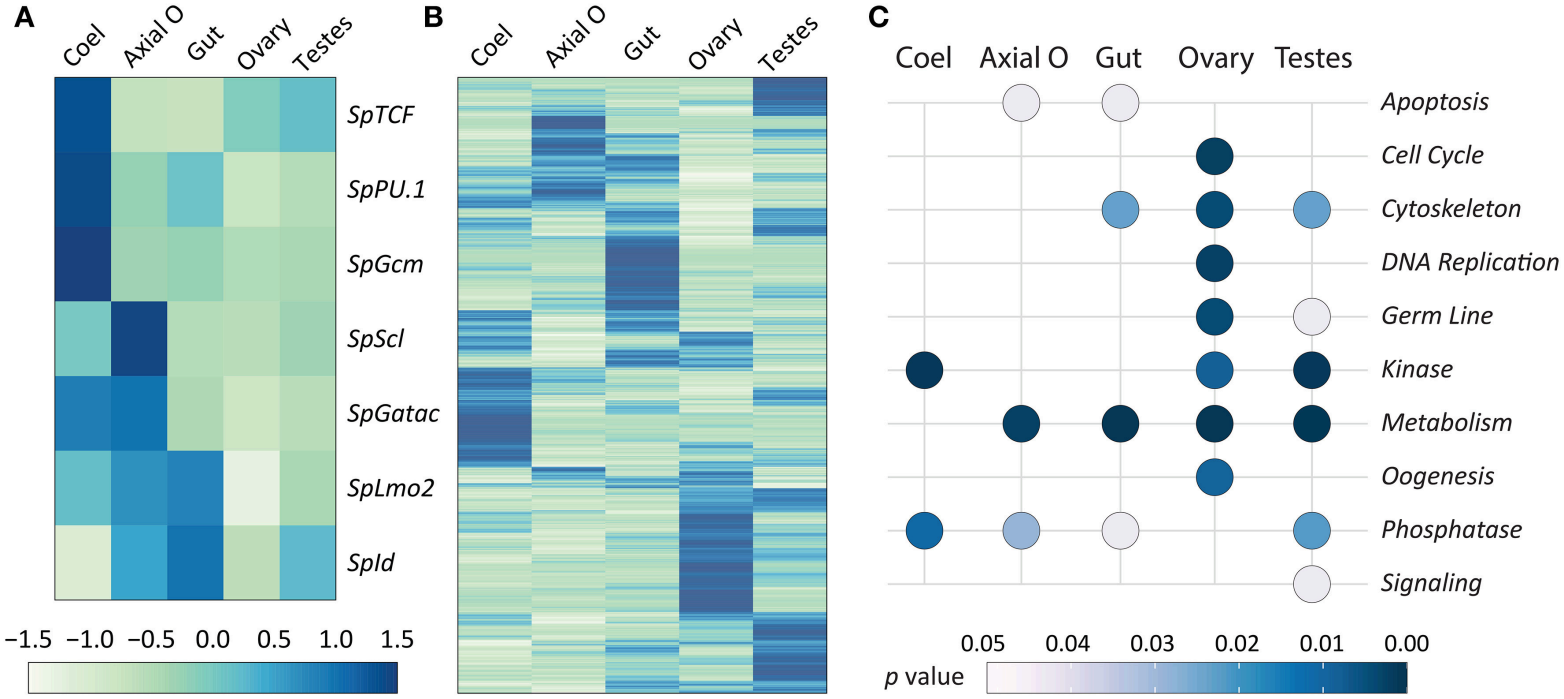
Expression of genes encoding transcription factors that control hematopoiesis are elevated in the axial organ and coelomocytes. The RNAseq dataset for adult sea urchin tissues (72) was used to analyze gene expression. Tissues were isolated from sea urchins that were not immune challenged. The pharynx was not included in the original dataset. **(A)** Differential expression levels of genes encoding transcription factors that function in the hematopoiesis GRN, based on results from embryos and larvae, were selected from the RNAseq dataset. Results show that different transcription factors may function in coelomocytes (Coel) compared to the axial organ (Axial O). Expression is generally lower in ovary and testes. **(B)** Differential expression of all genes is indicated for each tissue, which is based on read counts per transcript matching to the genome. The different tissues tend to express different sets of genes. Elevated gene expression is indicated in **(A,B)** by darker blue colors and diminished expression is in lighter shades of green. **(C)** Gene expression for the tissues is shown relative to predicted functions based on gene ontology (see Table 1 and Data Sheet 1). Significant association with function (*p* ≤ 0.05) is indicated by light gray, whereas all darker blue shades indicate non-significant association.

In addition to using qPCR to quantify the expression of transcription factor genes, publicly available RNAseq data for adult sea urchin tissues (72) were used to verify the qPCR results. RNAseq analysis was also employed to provide predictions of tissue functions based on insights from global gene expression. Transcriptome data are available for coelomocytes, axial organ, ovary, testes, and gut, but the pharynx was not evaluated. It should be noted that the RNAseq data were acquired from sea urchins that were not immune activated experimentally. RNAseq reads were mapped to the *S. purpuratus* genome (v4.2; www.echinobase.org), measured as counts per million per kilobase transcript [CPKM; (88)], and normalized across tissues (Figure 7). In the axial organ, *SpScl, SpLmo2*, and *SpId* transcripts were elevated relative to the other tissues (Figure 7A), which was generally consistent with the qPCR results (Figure 6). Notably, *SpGatac*, which is associated with maintaining pluripotency of immune cell precursors in both vertebrates as well as sea urchin embryos (59), was expressed at similar levels in coelomocytes and axial organ. *SpLmo2* and *SpId* were also expressed in the gut tissue. In contrast, very little expression of these transcription factor genes was apparent in the ovary and testes (Figure 7A). Although adult tissues often harbor either resident or circulating coelomocytes (e.g., *SpTrf*^+^ coelomocytes in the axial organ and gut (Figures 5A,D) (29), the distinct expression profiles between coelomocytes and the other tissues suggested that this contamination did not greatly influence the results.

A global analysis of transcript levels from the RNAseq data provided more detailed predictions regarding potential functions of the adult sea urchin tissues. Results indicated that different sets of genes showed elevated expression in different tissues (Figure 7B). These patterns of gene expression were evaluated through an expanded gene ontology (GO) analysis. To reduce spurious results that often interfere with analyzing expression data in non-model organisms, a custom ontology set was used, which was derived from manually curated gene sets (72). Although this gene set is partial, the annotations are likely to be more accurate because manual annotation was completed by experts in the field (78). Genes were divided into 24 primary categories and 132 subcategories based on two additional levels. For each tissue, a list of expressed transcripts was generated based on whether the RPKM was greater than the median expression level across all transcripts and tissue types in this study. *p* values for each GO annotation term were determined using hypergeometric tests. The axial organ showed significant enrichment of transcripts (*p* < 0.05) in 38 of the GO categories that included apoptosis, adhesion, ETS transcription factors, and several immunity and signaling categories (Figure 7C; Table 1; Data Sheet 1). Overall, results for gene expression based on either qPCR or RNAseq suggested that both the axial organ and the pharynx may be sites of hematopoiesis and the differentiation of all types of coelomocytes. Furthermore, the RNAseq results suggested that the axial organ may also function in the removal and recycling of senescent coelomocytes and foreign cells, which agreed with some older reports (36, 45, 89).

**Table 1:** A range of GO categories associate with the gene expression in the sea urchin axial organ^a^.

**GO Category^b^**	**Axial O^c^**	**Coel**	**Gut**	**Ovary**	**Testes**
Adhesion: Adhesion ECM Proteoglycan	0.009	0.018	0.130	0.029	0.076
Adhesion: Adhesion Receptor Cadherin	0.012	0.985	0.091	0.624	0.334
Apoptosis	0.014	0.028	0.012	0.754	0.170
Apoptosis: Apoptosis Bcl2	0.041	0.007	0.233	0.034	0.054
Cytoskeleton: Cytoskeleton Actin	0.035	0.058	0.000	0.000	0.001
Cytoskeleton: Cytoskeleton Actin: Cytoskeleton Actin Binding	0.040	0.135	0.001	0.003	0.002
Cytoskeleton: Cytoskeleton Dynein	0.029	0.041	0.170	0.023	0.001
Cytoskeleton: Cytoskeleton Tubulin	0.041	0.054	0.071	0.136	0.054
Defensome: Defensome Antioxidant	0.046	0.003	0.027	0.000	0.064
Defensome: Defensome HSP	0.007	0.263	0.018	0.023	0.012
GTPase: GTPase Ras	0.010	0.001	0.000	0.051	0.031
GTPase: GTPase Ras: GTPase Ras Arf	0.030	0.118	0.070	0.151	0.118
GTPase: GTPase Ras: GTPase Ras Rab	0.039	0.000	0.000	0.093	0.017
Immunity: Immunity Signal	0.001	0.000	0.006	0.529	0.044
Kinase	0.026	0.000	0.154	0.002	0.000
Kinase: Kinase AGC	0.003	0.017	0.276	0.006	0.046
Kinase: Kinase TK	0.048	0.018	0.224	0.795	0.238
Signaling: Signaling TGFB	0.000	0.121	0.004	0.022	0.000
Signaling: Signaling TGFB: Signaling TGFB Ligand	0.046	0.537	0.612	0.797	0.167
Signaling: Signaling_TGFB: Signaling TGFB Smad Interact	0.001	0.135	0.014	0.010	0.002
TF: TF Ets	0.001	0.000	0.020	0.120	0.013

a *p values determined by hypergeometric tests indicate significant association of the GO term with gene expression in a tissue*.

b *GO terms are selected based on significant association with the axial organ*.

c *Axial O, axial organ; Coel, Coelomocytes*.

## Discussion

### Maintenance of Coelomocyte Populations

The significant increase in the concentration of coelomocytes in the CF of IQ sea urchins in response to *V. diazotrophicus* or CF depletion is consistent with responses to injections of LPS or sham controls (23, 30). However, EdU uptake indicates that only about 10% of the coelomocytes in the CF are newly proliferated, suggesting that most of the cells enter the CF from unknown locations. These locations may be any tissue or organ because SpTrf^+^ phagocytes, and likely all other types of coelomocytes, are present in all major sea urchin tissues (29). Although most of the newly proliferated coelomocytes are polygonal phagocytes, within phagocyte subtypes the small and medium phagocytes show the greatest proliferation. These increases may be significant with regard to the sea urchin response to immune challenge because these two types of phagocytes show the highest expression of the anti-pathogen SpTrf proteins (10, 26) that is also consistent with increases in the percentages of SpTrf^+^ small phagocytes in response to immune challenge (23).

The presence of proliferated coelomocytes in the CF 75 days after a single EdU injection suggests a very slow rate of cell turnover in keeping with the slow appearance of proliferated cells that has implications for a long-lived population of cells. This is consistent with the hypothesis that coelomocytes proliferate in the hematopoietic tissues and are stored either in the same tissue or in other tissues until an immune or stress signal induces their release into the CF. Similarly, sessile hemocytes have been described for adult *Drosophila* (7, 90) although little information is available for other invertebrates. A slow turnover rate of coelomocytes is also consistent with the notion that newly proliferated cells may remain in the CF until they perform an immune function such as phagocytosis or encapsulation (perhaps to clear a microbial infection) and are subsequently removed from the CF. In adult sea urchins, coelomocyte concentrations decrease significantly within 5–6 h of bacterial challenge, and rebound to about starting levels by 24 h (11, 12). A similar but delayed pattern was noted here one day after injections of both heat-killed *V. diazotrophicus* and aCF (Figure 1B). It is not known whether this initial decrease is due to apoptosis of the coelomocytes that are involved in pathogen clearance [as has been reported for molluscs (91)], from encapsulation of aggregated bacteria and subsequent clearance of aggregates composed of bacteria and coelomocytes, from injury induced clot formation, or perhaps all of these possibilities acting simultaneously.

### The Axial Organ as a Site of Hematopoiesis

Speculations as to the hematopoietic source of coelomocytes in sea urchins and other echinoderms have suggested a range of tissues including the coelomic epithelium, Tiedemann's bodies, and the axial organ (13, 36–38, 67, 92). The axial organ has been proposed as a candidate site for hematopoiesis based on the richness of coelomocytes in this tissue (33, 35). BrdU uptake in the sea star, *Asterias rubens*, responding to injected LPS or concanavalin A suggests that the coelomic epithelium and Tiedemann's bodies are also sites of coelomocyte proliferation in asteroids (67), however, these tissues were not evaluated in this study and therefore cannot be eliminated as sources of coelomocytes in echinoids. That the axial organ may be a site of cell proliferation in *S. purpuratus* is consistent with elevated expression of *SpTie1/2* in the axial organ, in coelomocytes, and in sea urchin embryos when larval immune cells differentiate during gastrula to early pluteus stages (93). This also agrees with the RNAseq dataset (72) showing that transcripts from *SpTie* (SPU_026748 and SPU_024044) and *SpTie*-like genes (SPU_014858 and SPU_002763; www.echinobase.org) are elevated in the axial organ and in coelomocytes. In sea urchins, the SpTie homologs may have similar functions as the vertebrate receptors, which show hematopoiesis activities in adult mice (94). Our results indicate that the proportion of proliferated cells remains constant among animals irrespective of experimental treatment and whether the animals are IQ or not. Non-IQ sea urchins in constant contact with microbial organisms in unfiltered sea water may maintain a state of immune responsiveness. A corollary to this finding is that there may be an upper limit to the rate of coelomocyte proliferation, turnover, and replacement that could not be exceeded by the experimental manipulations employed in this study. This illustrates the value of using IQ animals to analyze immune responsiveness in echinoids. Overall, results are consistent with the proliferation of coelomocytes and their subsequent steady-state release from the axial organ, which would show no observable change in the number of proliferated cells. In agreement with the scientific literature dating back to the early 1800s, the axial organ appears to be a site of hematopoiesis in echinoids.

### The Axial Organ as a Site of Cell Recycling and Apoptosis

Although the axial organ in sea urchins has been speculated to have a wide range of functions (38, 39, 41, 43, 45), it also appears to be a collection site for disintegrating coelomocytes, including those involved in phagocytosis and encapsulation of foreign cells injected into the coelomic cavity, as well as clotted autologous coelomocytes, autologous sperm, sea star coelomocytes, the marine ciliate, *Uronema*, and inert foreign particles (34, 40, 44). In each case, the foreign material is encapsulated by coelomocytes and the aggregates are observed in the axial organ, which becomes enlarged and distended. In addition to removing foreign aggregates, the axial organ has also been suggested as a site for recycling senescent or functionally used cells (13, 34, 36, 37, 67). In agreement with these previous reports, the RNAseq analysis identifies genes that are upregulated in the axial organ and encode proteins associated with apoptosis. All results are consistent with predictions that the axial organ is a site of clearance and cellular recycling through apoptosis.

### The Pharynx as a Site of Hematopoiesis

Although the axial organ is the predicted site of hematopoiesis, the pharynx may also function as a hematopoietic tissue, which has not been considered or even mentioned in the published literature as of the date of this writing. Both the axial organ and the pharynx fulfill predictions for the ancestral animal hematopoietic tissue, which is an association with both a vascular system and coelomic spaces (50). Although echinoderms do not have a circulatory system for the purpose of gas exchange, a characteristic of the phylum is the water vascular system that is composed of fluid-filled vessels that function as a hydraulic system to extend the tube feet for locomotion and may enable gas exchange through the thin walls of the tube feet (95). The stone canal that is also part of the water vascular system and is associated with the madreporite on the aboral (dorsal) side of adult sea urchins, spans the central vertical axis of the animal, and supports the axial organ (Supplementary Figures 5A,B). At the top or dorsal end of Aristotle's Lantern, the stone canal connects to the ring canal of the water vascular system, which surrounds the aboral or dorsal end of the pharynx near the esophagus, and distributes fluid to five radial canals and into each tube foot. Both the axial organ and the pharynx are in contact with or are encased within coelomic spaces in regular echinoids (33). The axial organ is in direct contact with the central coelomic cavity and cells in the CF, and the pharynx is encased within the peripharyngeal cavity or sinus that is also fluid-filled. In addition to the pharyngeal walls that are typical columnar epithelia, five regions of connective tissue are peripheral to the pharynx and stabilize the mouth within the lantern (29, 33, 96). Within or near these regions of connective tissue, outside of the pharyngeal epithelium, is the location of many SpTrf^+^ cells (29) suggesting that this may be a site of coelomocyte proliferation.

### Expression of Transcription Factor Genes Is Consistent With Hematopoiesis in the Axial Organ and the Pharynx

Although sites of immune cell proliferation have been used to identify the hematopoietic tissues in many animals, efforts to employ this approach for sea urchins were unsuccessful. This outcome may have been due to a variety of factors including the genetic diversity among individual animals, which can mask significant differences among experimental groups of animals. Hence, the alternative approach of transcription factor gene expression analysis provides a more robust identification of the hematopoietic tissues. Both the axial organ and the pharynx express elevated levels of transcription factor genes that regulate hematopoiesis compared to expression levels in esophagus, gut, or gonad. None of these genes is expressed at particularly high levels, which is expected because the encoded transcription factors are not required in high concentrations, and they may only be expressed in a few hematopoietic stem cells and/or progenitor cells within these two tissues. *SpGcm* encodes the transcription factor that induces pigment cell differentiation in mesenchyme blastulae [(59, 60) reviewed in (16)] and is similarly expressed in both the axial organ and the pharynx. In blastula to larval stages, the continued expression of *SpGcm* plus the induction of *SpId* and other genes promote the pigment cell lineage, whereas the down regulation of *SpGcm*, de-repression of *SpGatac*, and expression of *SpScl* and *SpLmo2* drive differentiation and ingression of blastocoelar cells in late gastrulae. Both the axial organ and the pharynx show elevated expression of these transcription factor genes suggesting that hematopoiesis in the adult may follow a similar pattern in which a self-replicating hematopoietic stem cell may differentiate into at least two major lineages of cells. The elevated expression of other transcription factor genes, including *SpE2A, SpLmo2, SpPU.1*, and *SpTCF* in the axial organ and particularly in the pharynx also suggests the involvement of these tissues in hematopoiesis.

The identification of the pharynx as a hematopoietic site has some anatomical parallels in other animals. In humans, lymphatic tissues such as the thymus, lymph nodes, and tonsils are present near, around, and within the pharynx (97). Hematopoietic tissues are also present near digestive tracts in many invertebrates such the crustaceans in which the lymph gland is located near both the stomach and the brain [(52, 53, 98); reviewed in (9)]. It is noteworthy that proliferated hemocytes from a tunicate are produced *in vitro* by pharyngeal explants suggesting that the pharynx is a site of hematopoiesis in protochordates (99). Although there are variations in the expression levels of the hematopoietic transcription factor genes in sea urchin tissues based on results from qPCR and RNAseq, in the absence of reagents to detect all categories of adult coelomocytes, both the axial organ and the pharynx are proposed as sites for production of all types of coelomocytes.

## Conclusion

The identification of the axial organ as the hematopoietic tissue agrees with speculations from the older literature citing the presence of coelomocytes to infer the production and/or removal of these cells (33, 34, 40, 44). The patterns of coelomocyte proliferation in the CF and the axial organ and expression of the *SpTrf* gene family in phagocytes (29) are all consistent with the axial organ as a hematopoietic tissue. Expression of the evolutionarily conserved transcription factor genes encoding proteins that regulate hematopoiesis further supports this conclusion. Gene expression results also suggest the pharynx as an additional site for hematopoiesis, which has not been considered previously. Investigations of the homologous GRN and the associated developmental mechanisms employed to evaluate hematopoiesis and to identify the hematopoietic tissue in adult sea urchins will impact our understanding of the evolution of the innate and adaptive immune systems. Whether the pharynx in the sea urchin has immunological parallels to lymphoid tissues associated with the vertebrate mouth, and whether the sea urchin axial organ has spleen-like function, will require additional investigations. The purple sea urchin is likely to be a pivotal organism for studies in comparative developmental immunology (100) because of the position of echinoderm phylum at the base of the deuterostomes and as a sister group to the chordates (101–103).

## Author Contributions

PG undertook the collection and analysis of cells and tissues from IQ animals. JR assisted with differential cell counts. LCS collected tissues from the non-IQ sea urchins, and CR sectioned, and analyzed the tissues. KB assisted with figures and evaluated the RNAseq dataset. PG, KB, and LCS wrote the manuscript with input from CR and JR.

### Conflict of Interest Statement

The authors declare that the research was conducted in the absence of any commercial or financial relationships that could be construed as a potential conflict of interest.

## Abbreviations

AC320: coelomocyte buffer with 320 mM sucrose
aCF: artificial coelomic fluid
BSA: bovine serum albumin
BV: body volume
CF: coelomic fluid
CMFSW-EH: calcium- and magnesium-free sea water with EDTA and HEPES
CPM: counts per million
DAPI, 4′: 6-diamidino-2-phenylindole dihydrochloride
E2A: transcription factor E2-Alpha
EDTA: ethylene diamine tetraacetic acid
EdU: ethynyl deoxyuridine
FITC: fluorescein isothiocyanate
Gata: transcription factors that bind to DNA at GATA sequences
Gcm: glial cells missing
GRN: gene regulatory network
Id: inhibitor of DNA-binding/differentiation proteins
IgG: immunoglobulin G
IQ: immunoquiescent
Lmo2: LIM domain only 2
LPS: lipopolysaccharide
NSM: non-skeletogenic mesenchyme
PBS: phosphate buffered saline
qPCR: quantitative reverse transcription polymerase chain reaction
Scl: T-cell acute lymphocytic leukemia protein 1
Sp: *Strongylocentrotus purpuratus*
SRCR: scavenger receptor cysteine rich
TCF: transcription factor/lymphoid enhancer-binding factor
*Trf*: *Transformer* genes.
