# Supplementary material for: The Axial Organ and the Pharynx Are Sites of Hematopoiesis in the Sea Urchin
Source: Front Immunol. 2019 Apr 25;10:870. doi: 10.3389/fimmu.2019.00870 (PMC6494969; doi:10.3389/fimmu.2019.00870)
Supplement: Supplementary file 2 [file Data_Sheet_2.docx]

The axial organ and the pharynx are sites of hematopoiesis in the sea urchin

Preethi Golconda, Katherine M. Buckley, Caroline R. Reynolds, Jennifer Romanello, L. Courtney Smith

Department of Biological Sciences, George Washington University, Washington DC, USA

**Supplementary Figures**

**
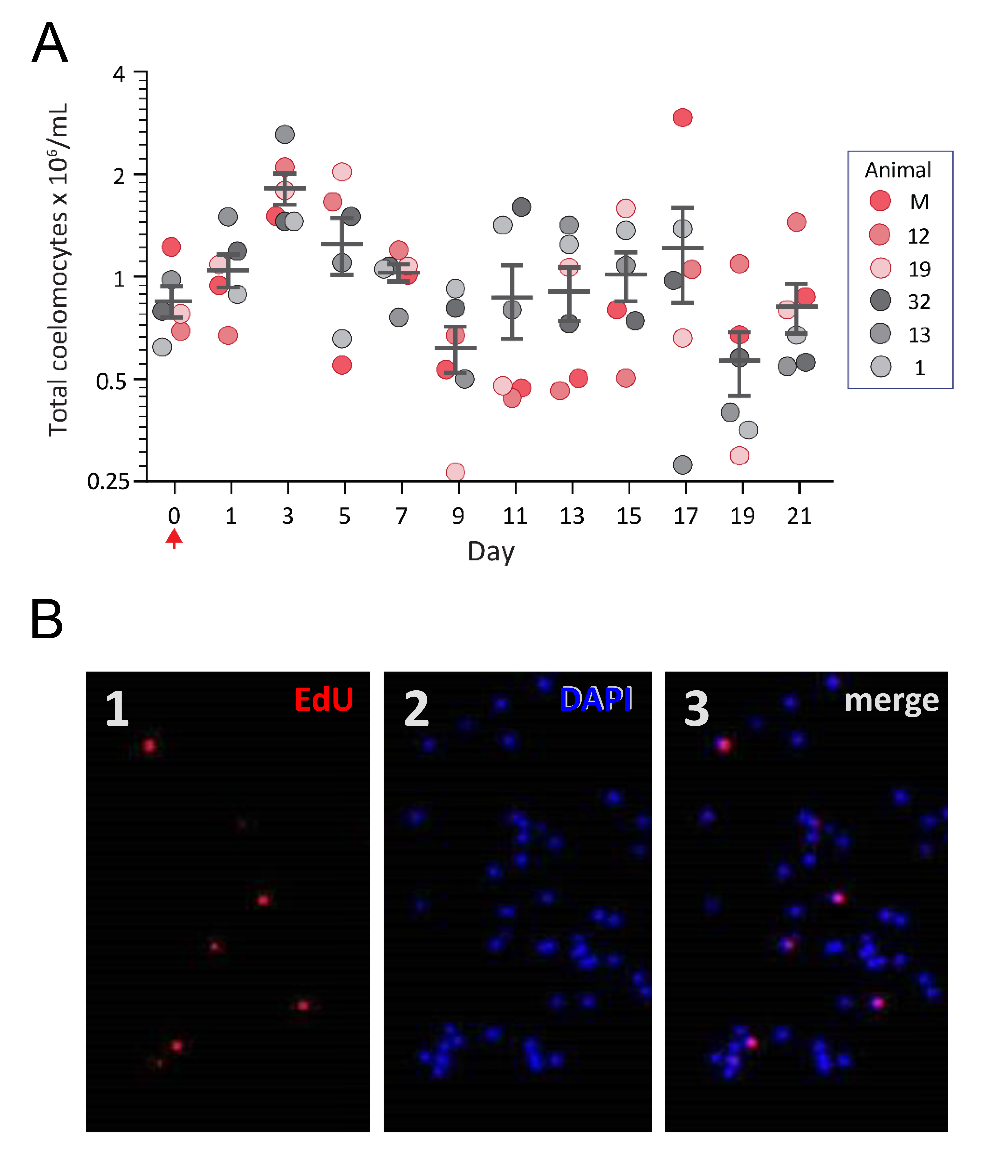
**

**Supplementary Fig. 1. A. Numbers of circulating coelomocytes do not change significantly over 21 days**. Total coelomocyte concentrations are shown for the animals that received a single injection (arrow) of EdU and either *Vibrio diazotrophicus* (animals M, 19, 12; pink dots) or aCF (sham injection; animals 32, 13, 1; gray dots). Although the numbers fluctuate over time, significant variations in cell concentration were not observed between the two groups of animals. Results for EdU uptake are shown in Fig. 1A in the main paper. **B. Proliferated phagocytes are identified by EdU incorporation into new DNA**. Fluorescent imaging of fixed phagocytes isolated from a sea urchin injected with EdU shows that EdU staining (**1**) can be used to identify the nuclei (**2**; DAPI) in newly proliferated phagocytes. The merge (**3**) of both EdU and DAPI staining shows nuclei of newly proliferated cells as pink.


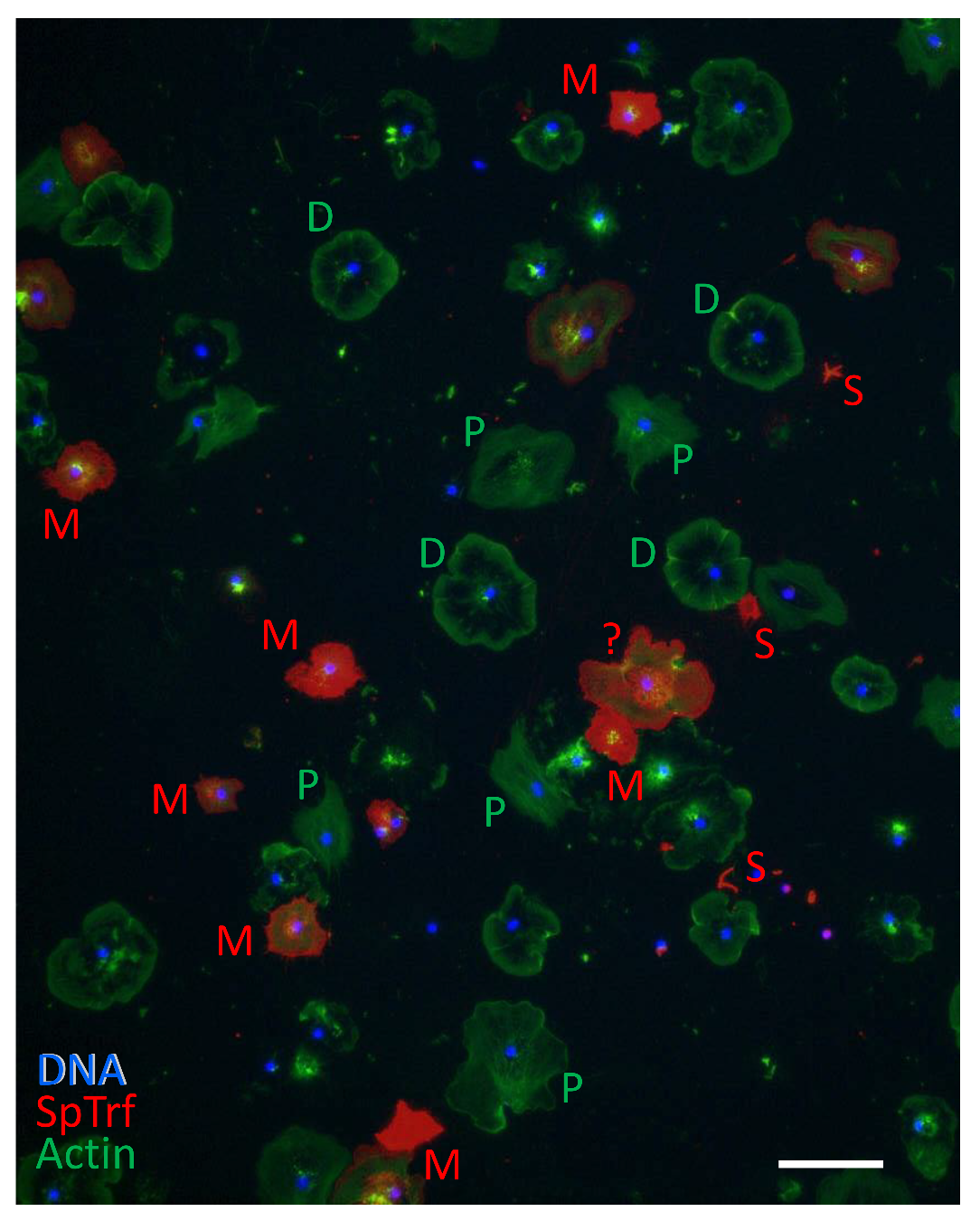


**Supplementary Fig. 2. Medium phagocytes are identified by their intermediate size and elevated level of SpTrf expression**. Glass-adherent phagocytes show elevated expression of SpTrf proteins in medium (M) and small (S) phagocytes that are differentiated by size and cellular morphology of lamellipodial vs. filopodial, respectively. The large phagocytes in this sample show typical structure of the actin cytoskeleton that differentiate these cells as discoidal (D) or polygonal (P). The low expression of SpTrf that is localized to vesicles in the large phagocytes is not obvious at this low magnification. The large SpTrf^+^ cell of unknown type (?) is similar to an SpTrf^+^ coelomocyte reported previously (see Fig. 2D in Ghosh et al. (2010) DCI 34:235-245). Scale bar indicates 40 μm.


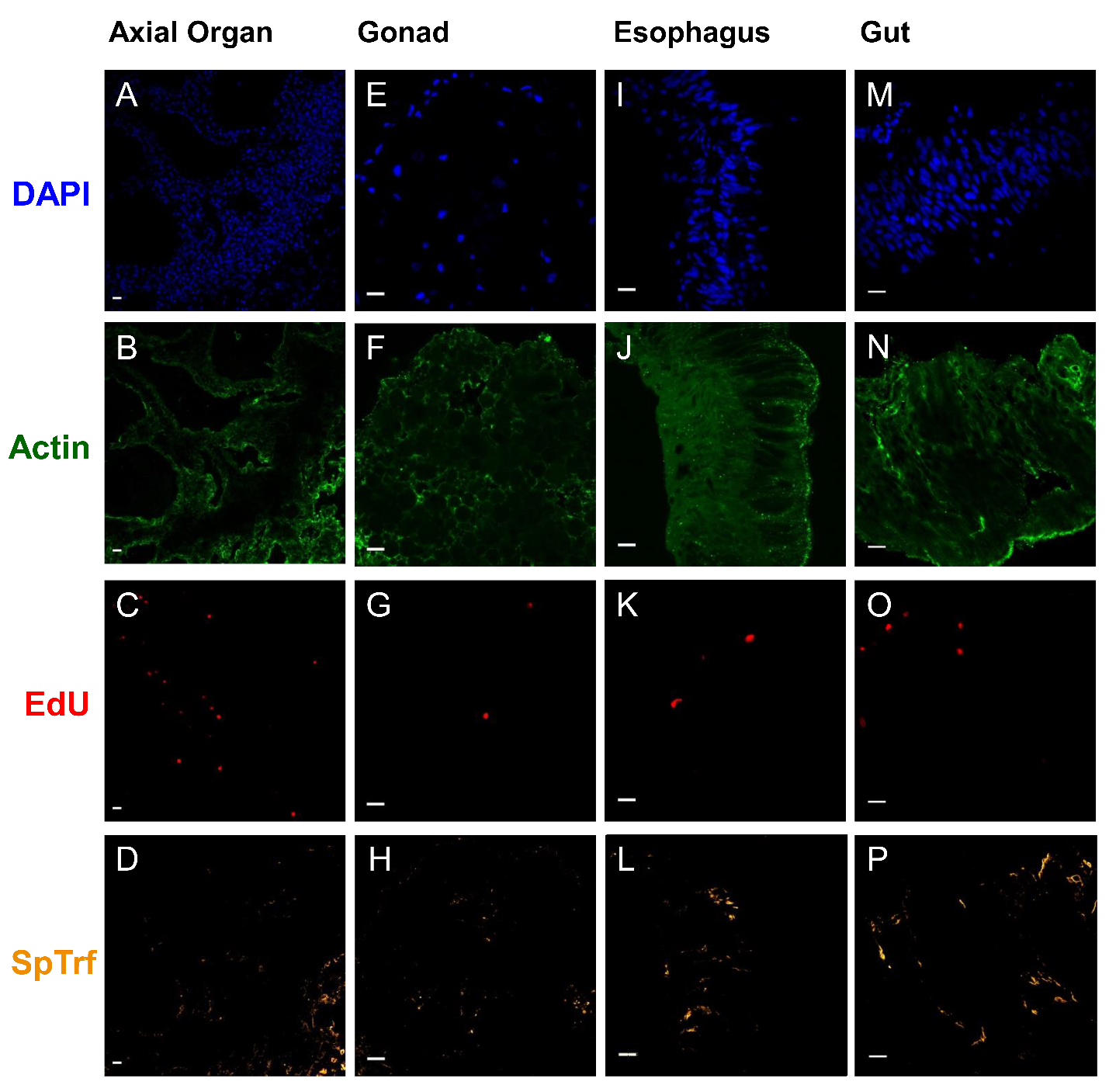


**Supplementary Fig. 3. These images of the axial organ, ovary, esophagus, and gut were used to generate the merged images in Fig. 5 in the main paper.** Sections are stained with DAPI to identify DNA in the nuclei (**A**, **E**, **I**, **M**), an anti-human actin monoclonal antibody to outline cells based on their cytoskeletons (**B**, **F**, **J**, **N**), processed for EdU incorporation to identify proliferated cells (**C**, **G**, **K**, **O**), and three rabbit anti-SpTrf antibodies (yellow) to identify phagocytes that express SpTrf (**D**, **H**, **L**, **P**). The scale bars indicate 10 μm.


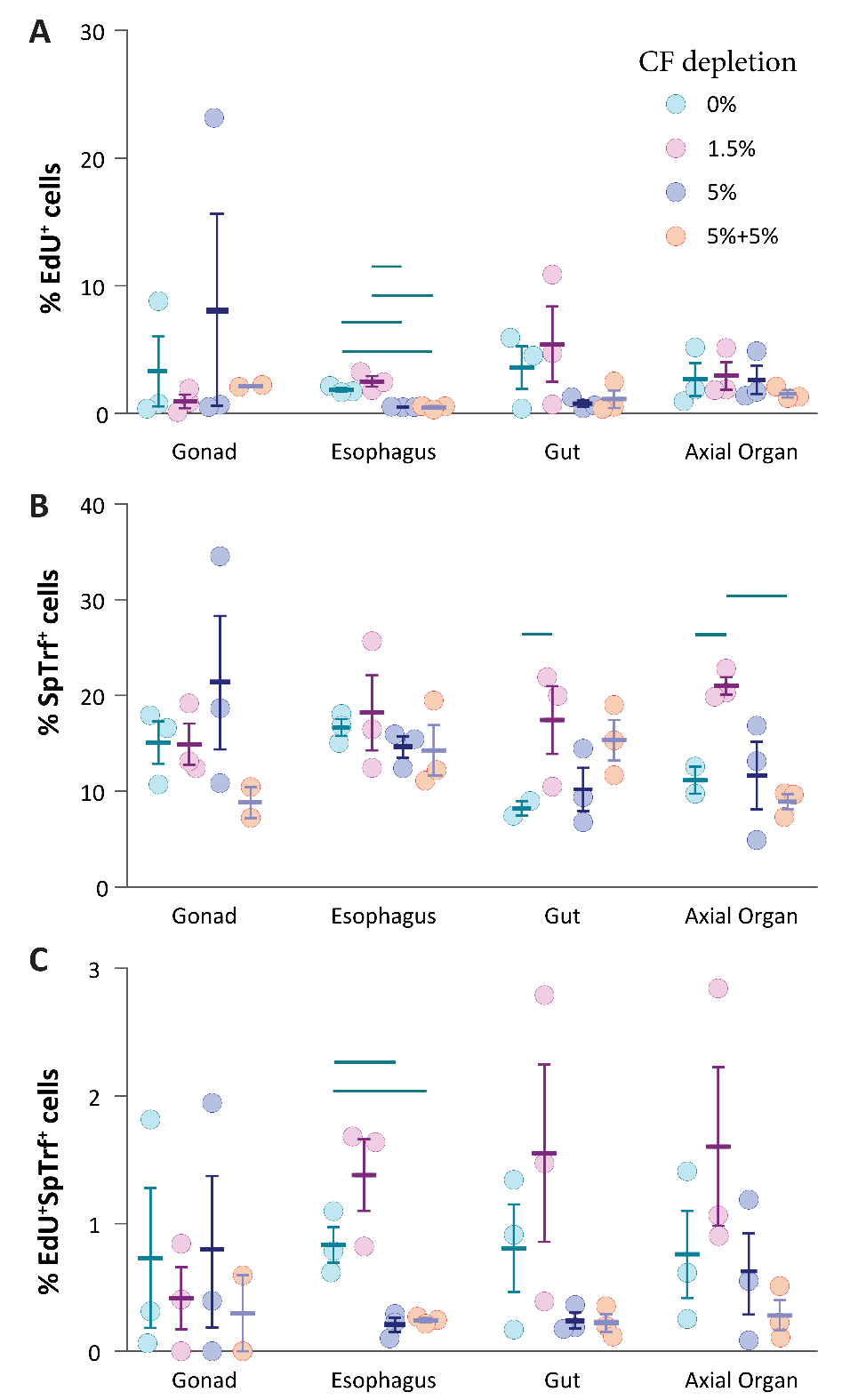


**Supplementary Fig. 4. EdU^+^ and SpTrf^+^ cells in tissues of animals after CF depletion.** Groups of sea urchins (n = 3) had varying percentages of CF depleted based on estimated body volume (BV). Tissues from each animal were fixed and sectioned, followed by evaluation for EdU and SpTrf (n = 3500 cells) and the percentage EdU^+^ cells, SpTrf^+^ cells, and EdU^+^/SpTrf^+^ cells were calculated. **A**. The percentages of EdU^+^ positive cells are shown for each tissue. **B**. The percentages of SpTrf^+^ cells are shown for each tissue. **C**. The percentages of cells that are both EdU^+^ and SpTrf^+^ are shown for each tissue. Horizontal lines among groups and within tissues indicate significant differences (*p* < 0.05).


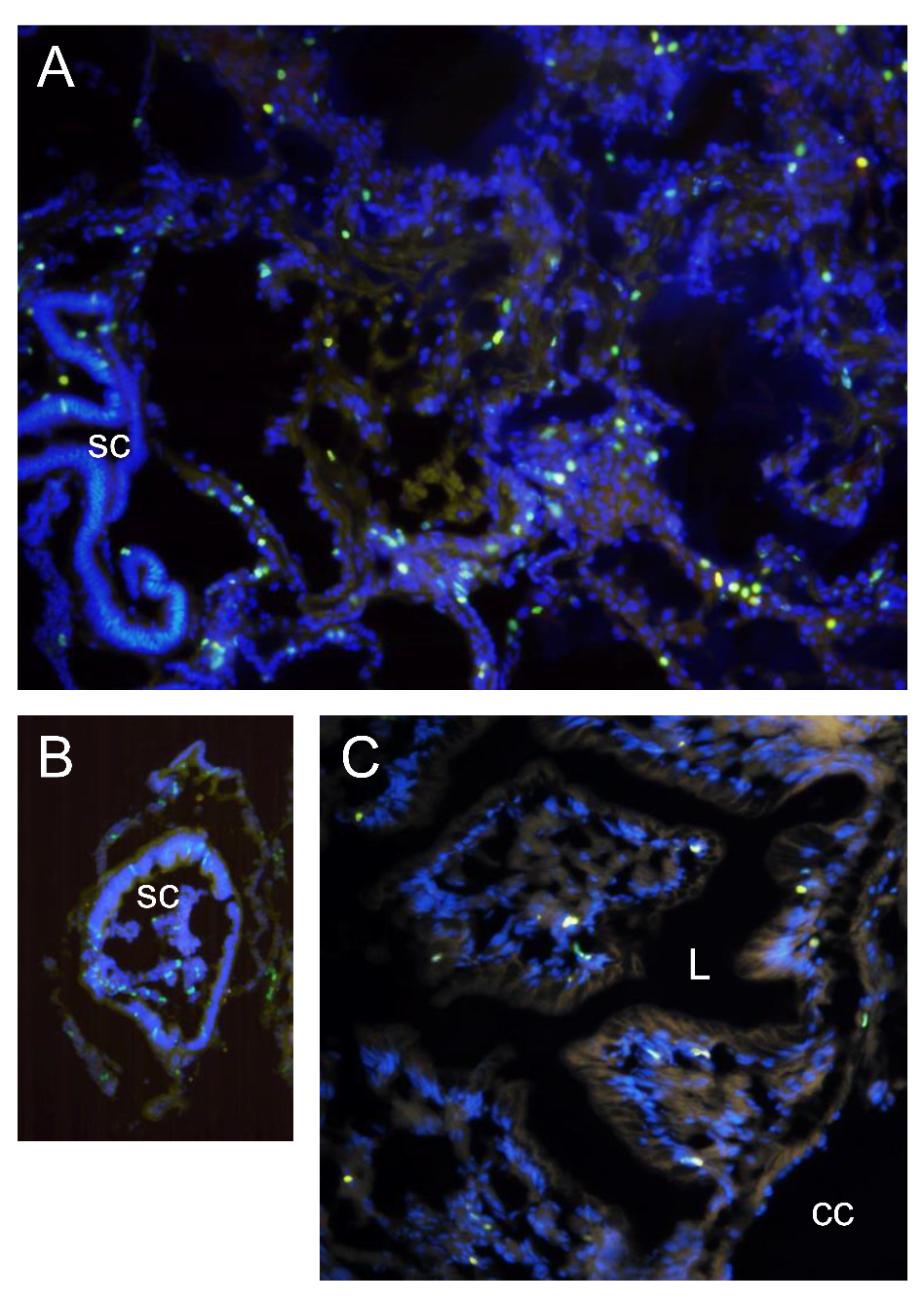


**Supplementary Fig. 5**. **The axial organ and esophagus contain EdU^+^ cells.** Tissues were collected from non-IQ sea urchins 22 days after injections with EdU. They were fixed, frozen, and sections were processed with the Alexa Fluor 488 ClickIt kit for EdU (Invitrogen) and counter stained with DAPI. Images were captured on an Axioskop fluorescent microscope (Zeiss) and an Infinity 3 color digital camera (Lumenera). Images were merged in Photoshop CC (Adobe) in which the green EdU^+^ nuclei were enhanced and the blue DAPI color was corrected. Between 4486 and 14805 nuclei were counted per tissue per group. **A**. EdU^+^ cells are distributed throughout the axial organ including the associated stone canal (sc). **B**. A cross section of a stone canal shows EdU^+^ cells. **C**. A cross section of an esophagus shows the coelomic cavity (cc) at the periphery of the image and the lumen (L) at the center. EdU^+^ nuclei are associated with the columnar epithelia and the basement membrane. These images were generated with guidance from Megan A. Barela Hudgell.
