# Supplementary material for: The Axial Organ and the Pharynx Are Sites of Hematopoiesis in the Sea Urchin
Source: Front Immunol. 2019 Apr 25;10:870. doi: 10.3389/fimmu.2019.00870 (PMC6494969; doi:10.3389/fimmu.2019.00870)
Supplement: Supplementary file 3 [file Data_Sheet_3.docx]

The axial organ and the pharynx are sites of hematopoiesis in the sea urchin

Preethi Golconda, Katherine M. Buckley, Caroline R. Reynolds, Jennifer Romanello, L. Courtney Smith

Department of Biological Sciences, George Washington University, Washington DC, USA

**Supplementary Tables**

**Supplementary Table 1.** Primer sequences

| **A. Primers for RT-PCR** | | | |  |
| --- | --- | --- | --- | --- |
| **Gene Name** | **Echinobase^1^ gene ID** | **Forward Primer** | **Reverse Primer** | **Annealing Temp (ºC)** |
| *SpL8* | SPU_010692 | ACCTCAAGGGCATCGTCAAG | TCATAGCAACACCACGCACTC | 55.0 |
| *SpGatac* | SPU_027015 | ACCGTCCCTTCTGCTGCTAC | GGCGTTACAGAGATAGTGACCG | 54.5 |
| *SpGcm* | SPU_006462 | CGAAAGGATGCCGAACAAG | TCTGTCGCTGTCCTCCATTG | 51.7 |
| *SpScl* | SPU_028093 | TAGCATCGGAGGACTTGGAAC | AGTGCGATTGGTCCGATTG | 53.0 |
| *SpTCF* | SPU_009520 | AGCAGCAGAGCAATGATAGGC | TTCCCTCCGTGGTCGTTCTC | 54.5 |
| *SpId* | SPU_015374 | ACCCTTACGAGACGCCTGAG | TGTTTGCATTTGTGGAGGTTG | 51.8 |
| *SpPU.1* | SPU_030060 | GACGCAGCAACTTCACAGGG | GGGCTTAATGTGTTGGACGATG | 53.8 |
| *SpE2A* | SPU_016343 | CCACAGTCATTATCGGAGAGGG | GACGCTCTTTCTCCTTCACCAC | 54.3 |
| *SpLmo2* | SPU_013569 | CGACTCCAATGGCGAAAC | AGTGGTCGTCGCACATGTC | 51.6 |
| **B. Primers for qPCR** | | |  |  |
| *SpL8* | SPU_010692 | CACAACAAGCACAGG | GCGTAGTCGATGGATCGGAGT | 59 |
| *SpGatac* | SPU_027015 | CAGGGACATCATGTGCAAAC | CCGTGTTTGAATGCCTTCTT | 52 |
| *SpGcm* | SPU_006462 | CGAAAGGATGCCGAACAAG | ACCGGTCCTTGGGCATTTC | 52 |
| *SpScl* | SPU_028093 | TAGCATCGGAGGACTTGGAAC | CACTATCATCCGCGTCCTCG | 54 |
| *SpTCF* | SPU_009520 | TGCGTTGCGGCCAGAGTAAG | CGATTCGCATGTGGTCCGTG | 56 |
| *SpId* | SPU_015374 | TTCACCATGTCCGATTGCTA | CGTCTGCAAGTCCTGGATGT | 52 |
| *SpPU.1* | SPU_030060 | GATACGGTGAAAAGCCATCG | TTCGCCAAAAGGAAAAACAC | 50 |
| *SpE2A* | SPU_016343 | AGGAGGCTACGCCAACCATC | AGGGTTGAGGTCCATGGCTG | 56 |
| *SpLmo2* | SPU_013569 | CTTGGAAGGAAGCTCTGCAA | AGGTGGTCATGACGAGTTCA | 53 |
| *SpTrf* | SPU_030144 | GGAACYGARGAMGGATCTC | AAATTCTACACCTCGGCGAC | ?? |

^1^www.echinobase.org/

**Supplementary Table 2**. Percentage of EdU^+^ phagocytes per subtype after CF depletion^1^

| **Phagocyte subtype** | **5% CF depletion**^2^ | **Day** | | | | | | |
| --- | --- | --- | --- | --- | --- | --- | --- | --- |
|  |  | **1** | **2** | **3** | **4** | **5** | **6** |  |
| Polygonal | Once | 2.3 | 3.7 | 6.5 | 13.1 | 19.0 | 22.4 |  |
|  | Twice | 1.4 | 2.1 | 8.9 | 18.9 | 5.0 | 12.5 |  |
| Discoidal | Once | 0.0 | 0.1 | 0.4 | 1.1 | 1.8 | 2.3 |  |
|  | Twice | 0.1 | 0.1 | 0.1 | 0.4 | 0.6 | 2.3 |  |
| Medium | Once | 5.0 | 29.7 | 14.5 | 56.4 | 40.4 | 20.0 |  |
|  | Twice | 42.8 | 24.3 | 64.3 | 29.9 | 7.0 | 40.0 |  |
| Small | Once | 19.6 | 57.7 | 57.7 | 65.3 | 60.5 | 65.5 |  |
|  | Twice | 34.4 | 36.0 | 48.2 | 61.9 | 52.2 | 49.2 |  |

^1^The percentage of EdU^+^ cells in each phagocyte class is based on the total number of phagocytes in each subtype.

^2^N = 6 for all groups of animals

**Supplementary Table 3.** Percentage of medium phagocytes in the CF^1^

|  | **Day^2^** | | | | | | |
| --- | --- | --- | --- | --- | --- | --- | --- |
| **Animal** | **0** | **1** | **2** | **3** | **4** | **5** | **6** |
| 2 | 11.4 | 16.0 | 16.4 | 18.3 | 16.5 | 17.3 | 14.4 |
| 4 | 6.2 | 58.2 | 54.0 | 30.3 | 26.4 | 7.9 | 27.8 |

^1^The percentage of medium phagocytes in two out-lier sea urchins is based on the total number of phagocytes in all classes.

^2^These two animals had 5% CF depletion on day 0 and day 3.

**Supplementary Table 4.** Cell proliferation in the axial organ, esophagus, and coelomocytes is not altered by immune challenge or injury in non-IQ sea urchins

| Tissue | Group^1^ | Percentage (SD) of EdU^+^ cells in individual animals | | | | | | Mean % (SD) per group | Mean % (SD) per tissue | Range of % proliferated cells |
| --- | --- | --- | --- | --- | --- | --- | --- | --- | --- | --- |
| Axial Organ | 1 | 5.81 (3.66) | | | 9.98 (1.52) | | | 6.14 (3.52) | 5.65 (3.27) | 0.29-12.6 |
|  | 2 | 4.89 (2.63) | | | 4.85 (2.05) | | | 4.87 (2.21) |  |  |
|  | 3 | 1.53 (1.09) | | | 6.76 (1.94) | | | 4.39 (3.13) |  |  |
| Esophagus | 1 | 4.83 (2.50) | | | 6.54 (2.01) | | | 5.68 (2.32) | 4.35 (3.04) | 0.37-12.3 |
|  | 2 | 4.64 (2.33) | | | 2.06 (0.57) | | | 3.56 (2.2) |  |  |
|  | 3 | 0.51 (0.12) | | | 6.88 (3.09) | | | 4.23 (3.99) |  |  |
| Coelomocytes^2^ | 1 | 9.53 | | | 26.02 | | | 17.77 (11.66) | 18.40 (9.25) | 5.17-35.94 |
|  | 2 | 23.51 | | 35.94 | | 18.22 | | 25.89 (9.10) |  |  |
|  | 3 | 5.17 | 15.87 | | 18.71 | | 12.65 | 13.10 (5.84) |  |  |

^1^Group 1; Sea urchins were injected with EdU and *V. diazotrophicus* on days 0, 3, 6, 21.

Group 2; sea urchins were injected with EdU and *V. diazotrophicus* on days 0, 3, and 6. On day 21, they were injected with aCF.

Group 3; sea urchins were injected with aCF on days 0, 3, 6, 21.

^2^Coelomocytes were collected from all animals on day 21 and analyzed for EdU incorporation by flow cytometry.

All animals were sacrificed on day 22 for collection of the axial organ and esophagus. EdU^+^ cells were evaluated in sections by microscopy and counted manually.

**Supplementary Table 5**. Single-factor ANOVA *p* values indicate no differences in gene expression within tissues among most groups of animals after CF depletion^1^

| Tissue | Genes encoding transcription factors that function in the hematopoiesis GRN | | | | | | | | SpTrf |
| --- | --- | --- | --- | --- | --- | --- | --- | --- | --- |
|  | *SpE2A* | *SpTCF* | *SpPU.1* | *SpId* | *SpLmo2* | *SpGatac* | *SpGcm* | *SpScl* |  |
| Axial organ^2^ | 0.0690 | 0.0909 | 0.0252^3^ | 0.08056 | 0.0203^3^ | 0.03494^3^ | 0.0204^3^ | 0.02041^3^ | 0.2475 |
| Pharynx | 0.3896 | 0.2395 | 0.2450 | 0.41989 | 0.6699 | 0.54280 | 0.5669 | 0.4833 | 0.3449 |
| Esophagus | 0.5998 | 0.8055 | 0.6559 | 0.46920 | 0.6527 | 0.48236 | 0.7105 | 0.8686 | 0.9414 |
| Gut | 0.3293 | 0.1610 | 0.2901 | 0.27507 | 0.6835 | 0.34704 | 0.3922 | 0.9300 | 0.8084 |
| Gonad | 0.4800 | 0.5509 | 0.7644 | 0.60630 | 0.5427 | 0.47985 | 0.6614 | 0.7573 | 0.6261 |

^1^Animals were depleted of CF based on estimated body volume (BV). One group was depleted of CF equal to 5% of the esitmated BV, and another group was depleted of 5% twice. Controls were not depleted of CF.

^2^The expression level of the control gene, *SpL8*, in one of three control animals (0% CF depletion) for the axial organ was identified as an outlier based on τ and Q statistical tests. Hence, that data point was omitted from the analysis of gene expression.

^3^Gene expression is significantly higher in the group from which 5% of the CF was depleted once.

**Supplementary Table 6**. Unpaired *t*-test *p* values indicate differences in gene expression among tissues

| ***SpE2A*** | Axial Organ | Pharynx | Esophagus | Gut | Gonad |
| --- | --- | --- | --- | --- | --- |
| Pharynx | 0.0505 |  |  |  |  |
| Esophagus | 0.1385 | 0.0070 |  |  |  |
| Gut | 0.0461 | 0.0041 | 0.0188 |  |  |
| Gonad | 0.1872 | 0.0116 | 0.9421 | 0.1207 |  |
| Coelomocytes | 0.0997 | 0.0112 | 0.0875 | 0.1656 | 0.2860 |
| ***SpTCF*** | Axial Organ | Pharynx | Esophagus | Gut | Gonad |
| Pharynx | 0.4038 |  |  |  |  |
| Esophagus | 0.1189 | 0.0067 |  |  |  |
| Gut | 0.0814 | 0.0041 | 0.0304 |  |  |
| Gonad | 0.6358 | 0.1253 | 0.0461 | 0.0196 |  |
| Coelomocytes | 0.0957 | 0.0076 | 0.0005 | 0.0114 | 0.0262 |
| ***SpPU.1*** | Axial Organ | Pharynx | Esophagus | Gut | Gonad |
| Pharynx | 0.0177 |  |  |  |  |
| Esophagus | 0.1798 | 0.0021 |  |  |  |
| Gut | 0.1320 | 0.0018 | 0.6650 |  |  |
| Gonad | 0.3810 | 0.0056 | 0.5494 | 0.3688 |  |
| Coelomocytes | 0.7318 | 0.0925 | 0.3886 | 0.3643 | 0.4577 |
| ***SpId*** | Axial Organ | Pharynx | Esophagus | Gut | Gonad |
| Pharynx | 0.3958 |  |  |  |  |
| Esophagus | 0.0353 | 0.0029 |  |  |  |
| Gut | 0.0189 | 0.0015 | 0.0338 |  |  |
| Gonad | 0.0396 | 0.0041 | 0.5616 | 0.4365 |  |
| Coelomocytes | 0.0356 | 0.0041 | 0.0450 | 0.4587 | 0.2567 |
| ***SpLmo2*** | Axial Organ | Pharynx | Esophagus | Gut | Gonad |
| Pharynx | 0.0426 |  |  |  |  |
| Esophagus | 0.0888 | 0.0097 |  |  |  |
| Gut | 0.0233 | 0.0233 | 0.0511 |  |  |
| Gonad | 0.1744 | 0.1744 | 0.7332 | 0.1176 |  |
| Coelomocytes | 0.0487 | 0.0143 | 0.0596 | 0.9738 | 0.1578 |
| ***SpGatac*** | Axial Organ | Pharynx | Esophagus | Gut | Gonad |
| Pharynx | 0.0476 |  |  |  |  |
| Esophagus | 0.0212 | 0.0076 |  |  |  |
| Gut | 0.0033 | 0.0049 | 0.0456 |  |  |
| Gonad | 0.1633 | 0.0126 | 0.9665 | 0.2516 |  |
| Coelomocytes | 0.5117 | 0.0316 | 0.2680 | 0.1550 | 0.2872 |
| ***SpGcm*** | Axial Organ | Pharynx | Esophagus | Gut | Gonad |
| Pharynx | 0.1181 |  |  |  |  |
| Esophagus | 0.0857 | 0.0089 |  |  |  |
| Gut | 0.0381 | 0.0055 | 0.0096 |  |  |
| Gonad | 0.1541 | 0.0171 | 0.6484 | 0.1006 |  |
| Coelomocytes | 0.0923 | 0.0146 | 0.2324 | 0.1638 | 0.3266 |
| ***SpScl*** | Axial Organ | Pharynx | Esophagus | Gut | Gonad |
| Pharynx | 0.2537 |  |  |  |  |
| Esophagus | 0.0054 | 0.0080 |  |  |  |
| Gut | 0.0024 | 0.0055 | 0.0848 |  |  |
| Gonad | 0.0177 | 0.0171 | 0.3646 | 0.0408 |  |
| Coelomocytes | 0.0156 | 0.0152 | 0.7645 | 0.1553 | 0.2731 |
| ***SpTrf*** | Axial Organ | Pharynx | Esophagus | Gut | Gonad |
| Pharynx | 0.9683 |  |  |  |  |
| Esophagus | 0.0086 | 0.0030 |  |  |  |
| Gut | 0.0031 | 0.0008 | 0.0271 |  |  |
| Gonad | 0.0150 | 0.0061 | 0.8680 | 0.2647 |  |
| Coelomocytes | 0.0163 | 0.0044 | 0.2682 | 0.3650 | 0.6056 |

**Supplementary Table 7**. Single-factor ANOVA *p* values indicate significant differences in gene expression among tissues

| CF depletion treatments^1^ | Genes encoding transcription factors that function in the hematopoiesis GRN | | | | | | | |  |
| --- | --- | --- | --- | --- | --- | --- | --- | --- | --- |
|  | *SpE2A* | *SpTCF* | *SpPU.1* | *SpID* | *SpLmo2* | *SpGatac* | *SpGcm* | *SpScl* | *SpTrf* |
| 0%, 5%, 5%+5% | 1.43e-4 | 9.11e-3 | 3.71e-5 | 9.85e-5 | 1.93e-4 | 1.32e-4 | 5.5e-4 | 2.22e-4 | 6.77e-5 |
| 0% and 5% | 5.34e-4 | 2.17e-2 | 2.66e-4 | 1.75e-3 | 7.85e-4 | 8.48e-4 | 2.16e-3 | 2.95e-3 | 4.05e-5 |
| 0% | 5.52e-4 | 1.15e-4 | 4.22e-2 | 2.85e-3 | 2.57e-3 | 5.40e-4 | 7.67e-4 | 3.10e-3 | 3.09e-3 |
| 5% | 4.21e-3 | 5.68e-2 | 1.91e-3 | 2.19e-2 | 3.83e-2 | 4.20e-2 | 2.81e-2 | 4.03e-2 | 1.22e-2 |
| 5% +5% | 4.27e-1 | 3.18e-1 | 3.0e-1 | 7.44e-2 | 4.50e-1 | 4.17e-1 | 4.12e-1 | 2.09e-1 | 3.83e-1 |

^1^Animals were depleted of CF based on estimated body volume (BV). One group was depleted of CF equal to 5% of the esitmated BV, and another group was depleted of 5% twice. Controls were not depleted of CF.

**Supplementary Table 8**. Bonferroni post test *p* values after two-factor, non-parametric ANOVA analysis indicate significant differences in gene expression in the axial organ and pharynx relative to other tissues^1^

| ***SpE2A*** | Signficance is due to differences between tissues and not between CF depletion groups | | | |
| --- | --- | --- | --- | --- |
|  | Axial Organ | Pharynx | Esophagus | Gut |
| Pharynx | < 0.05 |  |  |  |
| Esophagus | ns | < 0.05 |  |  |
| Gut | ns | < 0.05 | ns |  |
| Gonad | ns | < 0.05 | ns | ns |
| ***SpTCF*** | Signficance is due to differences between tissues and between CF depletion groups | | | |
|  | Axial Organ | Pharynx | Esophagus | Gut |
| Pharynx | ns |  |  |  |
| Esophagus | < 0.05 | < 0.05 |  |  |
| Gut | < 0.05 | < 0.05 | ns |  |
| Gonad | ns | ns | ns | ns |
| ***SpPU.1*** | Signficance is due to differences between tissues and between CF depletion groups | | | |
|  | Axial Organ | Pharynx | Esophagus | Gut |
| Pharynx | < 0.05 |  |  |  |
| Esophagus | ns | < 0.05 |  |  |
| Gut | ns | < 0.05 | ns |  |
| Gonad | ns | < 0.05 | ns | ns |
| ***SpId*** | Signficance is due to differences between tissues and between CF depletion groups | | | |
|  | Axial Organ | Pharynx | Esophagus | Gut |
| Pharynx | ns |  |  |  |
| Esophagus | < 0.05 | < 0.05 |  |  |
| Gut | < 0.05 | < 0.05 | ns |  |
| Gonad | < 0.05 | < 0.05 | ns | ns |
| ***SpLmo2*** | Signficance is due to differences between tissues and not between CF depletion groups | | | |
|  | Axial Organ | Pharynx | Esophagus | Gut |
| Pharynx | ns |  |  |  |
| Esophagus | ns | < 0.05 |  |  |
| Gut | ns | < 0.05 | ns |  |
| Gonad | ns | < 0.05 | ns | ns |
| ***SpGatac*** | Signficance is due to differences between tissues and not between CF depletion groups | | | |
|  | Axial Organ | Pharynx | Esophagus | Gut |
| Pharynx | < 0.05 |  |  |  |
| Esophagus | ns | < 0.05 |  |  |
| Gut | ns | < 0.05 | ns |  |
| Gonad | ns | < 0.05 | ns | ns |
| ***SpGcm*** | Signficance is due to differences between tissues and not between CF depletion groups | | | |
|  | Axial Organ | Pharynx | Esophagus | Gut |
| Pharynx | ns |  |  |  |
| Esophagus | ns | < 0.05 |  |  |
| Gut | ns | < 0.05 | ns |  |
| Gonad | ns | < 0.05 | ns | ns |
| ***SpScl*** | Signficance is due to differences between tissues and not between CF depletion groups | | | |
|  | Axial Organ | Pharynx | Esophagus | Gut |
| Pharynx | ns |  |  |  |
| Esophagus | < 0.05 | < 0.05 |  |  |
| Gut | < 0.05 | < 0.05 | ns |  |
| Gonad | < 0.05 | < 0.05 | ns | ns |
| ***SpTrf*** | Signficance is due to differences between tissues and not between CF depletion groups | | | |
|  | Axial Organ | Pharynx | Esophagus | Gut |
| Pharynx | ns |  |  |  |
| Esophagus | < 0.05 | < 0.05 |  |  |
| Gut | < 0.05 | < 0.05 | ns |  |
| Gonad | < 0.05 | < 0.05 | ns | ns |

^1^Gene expression for coelomocytes was omitted because samples were only evaluated prior to CF depletion.
